# The MASLD–Cardio-Oncology Triangle: Dietary Patterns, Metabolic Remodelling and Implications for Cancer Therapy Tolerance

**DOI:** 10.3390/nu18152527

**Published:** 2026-08-04

**Authors:** Francesca La Rocca, Graziella Privitera, Calogero Geraci, Valentina Morello, Giulio Geraci, Valentina Paternò, Ciro Santoro, Roberta Esposito

**Affiliations:** 1 Department of Clinical and Experimental Medicine, University of Catania, 95122 Catania, Italy; francescalarocca9401@gmail.com (F.L.R.); graziella.privitera@unict.it (G.P.); 2 Cardiobesity Group, 93100 Caltanissetta, Italy; cgeraci81@gmail.com; 3 Cardiology Unit, Azienda Sanitaria Provinciale of Caltanissetta (ASP), 93100 Caltanissetta, Italy; 4 Indipendent Nutrition Biologist, 93100 Caltanissetta, Italy; valentinamorello86@gmail.com; 5 Department of Medicine and Surgery, “Kore” University of Enna, 94100 Enna, Italy; giulio.geraci@unikore.it (G.G.); valentinapaterno91@gmail.com (V.P.); 6 Department of Pharmacy Health and Nutritional Science, University of Calabria, 87036 Rende, Italy; cirohsantoro@gmail.com; 7 Department of Clinical Medicine and Surgery, Federico II University Hospital, 80131 Naples, Italy

**Keywords:** MASLD, MASH, cardio-oncology, cardiotoxicity, Mediterranean diet, precision nutrition, mitochondrial dysfunction, NLRP3 inflammasome

## Abstract

**Background:** Metabolic dysfunction-associated steatotic liver disease (MASLD) is highly prevalent worldwide and represents the hepatic manifestation of a systemic cardiometabolic-inflammatory syndrome rather than an isolated organ disease. In parallel, anticancer therapies carry a well-recognised burden of cancer therapy-related cardiovascular toxicity (CTR-CVT). Evidence suggests that the metabolic-inflammatory cascade driving steatosis → steatohepatitis → fibrosis may also contribute to endothelial dysfunction, myocardial remodelling and cardiomyocyte vulnerability to chemotherapy-induced oxidative stress. This narrative review proposes a unifying conceptual framework in which MASLD may act as a potential amplifier of cardiotoxicity in oncology patients and examines whether lifestyle and dietary interventions could mitigate this cumulative risk. **Methods:** A structured literature search of PubMed, Scopus and Web of Science was performed, prioritising systematic reviews, meta-analyses, randomised controlled trials, large cohort studies and recent international guidelines on MASLD, cardio-oncology and nutritional interventions. **Results:** Four converging molecular axes were identified as plausible links between MASLD and cardiomyocyte susceptibility to anticancer therapy: mitochondrial dysfunction with reactive oxygen species overproduction, NLRP3 inflammasome activation and metaflammation, endothelial nitric oxide impairment, and pro-fibrotic TGF-β/hepatic stellate cell signalling. Mediterranean-style dietary patterns, selected micronutrients and emerging metabolic therapies modulate the same network and may offer translational opportunities. **Conclusions:** Reframing MASLD as a potentially modifiable amplifier of CTR-CVT supports the integration of hepatic phenotyping into baseline cardio-oncology risk stratification and the use of personalised nutrition as a precision tool acting on shared mitochondrial, inflammatory, endothelial and fibrotic pathways. Multidisciplinary framework and prospective interventional studies, adopting composite hepato-cardio-oncological endpoints, are warranted.

## 1. Introduction

Over the past three years, the conceptual boundaries between hepatology, cardiology and oncology have become progressively interconnected. The 2023 multisociety consensus replaced the historical term non-alcoholic fatty liver disease (NAFLD) with metabolic dysfunction-associated steatotic liver disease (MASLD), framing hepatic steatosis within an explicit cardiometabolic framework. MASLD is diagnosed when hepatic steatosis coexists with at least one of five cardiometabolic criteria: overweight or obesity, dysglycaemia or established type 2 diabetes, hypertension, hypertriglyceridaemia, or low high-density lipoprotein (HDL) cholesterol. Metabolic dysfunction-associated steatohepatitis (MASH) denotes the necroinflammatory phenotype of the disease [1]. The 2024 EASL-EASD-EASO Clinical Practice Guidelines on MASLD adopted this nomenclature as the reference framework for screening and risk stratification, recommending case-finding strategies, particularly in the presence of type 2 diabetes or obesity with additional metabolic risk factors [2,3].

The epidemiological burden underlying this conceptual shift is striking, positioning MASLD as one of the most prevalent chronic conditions of the twenty-first century and a critical determinant of population health. Current estimates indicate that its prevalence will continue to rise over the coming decades, potentially affecting more than half of the adult population by 2050, if current metabolic trends persist [4,5,6]. Clinically significant fibrosis (F2–F4) has been reported in around 20% of overweight or obese individuals with steatotic liver disease [7] and in more than one-third of those with type 2 diabetes [8]. Concurrently, recent cardiovascular–liver–metabolic recommendations, scientific statements and mechanistic reviews have converged on the need to interpret MASLD within a broader cardiometabolic and multisystem framework [9,10,11]. This perspective is supported by large cohort data in which excess mortality in MASLD is driven primarily by extrahepatic cancers, followed by cirrhosis and cardiovascular disease [12].

MASLD is increasingly recognised as a relevant and modifiable cardiovascular risk factor [9]. Epidemiological studies and meta-analyses have associated MASLD with major adverse cardiovascular events, cardiovascular mortality, heart failure and atrial fibrillation, with effect sizes increasing in parallel with the severity of liver disease, particularly fibrosis stage [13,14,15,16,17].

MASLD is also associated with a higher incidence of extrahepatic malignancies: reported risks are approximately 1.5- to 2-fold higher for gastrointestinal cancers and 1,2- to 1,5-fold higher for lung, breast, gynaecological and urinary tract cancers [18,19]. The contribution of MASLD and MASH to hepatocellular carcinoma is increasing, and MASLD has become the leading underlying aetiology of hepatocellular carcinoma in Sweden [19,20]. Mechanistically, this systemic burden may reflect a shared biological substrate involving insulin resistance, lipotoxicity, mitochondrial dysfunction, chronic low-grade inflammation, gut dysbiosis and profibrotic signalling [19,21,22,23]. However, these mechanistic pathways should be regarded as biologically plausible explanations for the observed associations rather than as definitive evidence of causality.

These epidemiological and pathophysiological trends may have important implications for oncology. A growing proportion of patients with cancer are older, overweight or obese, dysmetabolic, frequently MASLD-positive and potentially eligible for cardiotoxic systemic therapies, including anthracyclines, HER2-targeted agents, fluoropyrimidines, vascular endothelial growth factor (VEGF) or tyrosine kinase inhibitors, and immune checkpoint inhibitors. The 2022 European Society of Cardiology Guidelines on Cardio-Oncology emphasise that baseline cardiovascular status influences the subsequent risk of cancer therapy-related cardiovascular toxicity (CTR-CVT) and recommend pretreatment risk assessment to guide cardioprotective strategies and surveillance [24,25]. However, current Heart Failure Association-International Cardio-Oncology Society (HFA-ICOS) risk-stratification tools assess traditional cardiovascular risk factors, previous cardiovascular disease, biomarkers and cardiac imaging without routinely incorporating dedicated measures of hepatic steatosis or fibrosis, such as fibrosis-4 index (FIB-4) or liver stiffness [24,25]. Whether liver-related assessment provides incremental prognostic value in cardio-oncology remains uncertain. MASLD may identify a pre-existing metabolic and inflammatory substrate that could increase vulnerability to CTR-CVT. However, this concept remains a biologically plausible hypothesis that requires prospective clinical validation.

To date, MASLD, cardio-oncology and personalised nutrition have been addressed largely as parallel fields. Hepatology-focused reviews have documented the cardiovascular implications of MASLD without integrating oncological outcomes [9,19]; cardio-oncology guidelines and consensus documents have defined CTR-CVT risk stratification without incorporating hepatic phenotyping [24,25,26]; nutrition-focused reviews have examined Mediterranean-style dietary patterns and lifestyle interventions in cancer survivorship without integrating liver-specific assessment [27,28,29]. To the best of our knowledge, we identified no previous review that formally articulated MASLD as a shared systemic substrate across these three domains or proposed an operational framework in which non-invasive hepatic phenotyping is integrated into cardio-oncology risk stratification and personalised nutrition.

The present narrative review addresses this gap by proposing the MASLD–Cardio-Oncology Triangle as a working framework linking hepatic steatofibrosis, cardiovascular vulnerability, anticancer therapy and dietary exposures, within which dietary patterns and lifestyle interventions may represent modifiable entry points influencing metabolic resilience, cardiovascular vulnerability and, potentially, tolerance to anticancer therapy. The review therefore aims to: (i) outline the shared metabolic, inflammatory, endothelial and mitochondrial pathways linking MASLD progression to cardiomyocyte vulnerability during anticancer therapy; (ii) critically examine dietary patterns, gut–liver axis modulation, selected nutraceuticals and management of sarcopenic obesity as potential transversal modifiers of this substrate; and (iii) translate the framework into a multidisciplinary three-phase clinical pathway, encompassing pre-treatment baseline assessment, intra-treatment monitoring and post-treatment survivorship, that can be prospectively tested in future interventional studies.

## 2. Materials and Methods

This narrative review was based on a structured literature search conducted in PubMed (National Library of Medicine, Bethesda, MD, USA), Scopus (Elsevier B.V., Amsterdam, The Netherlands), and Web of Science Core Collection (Clarivate, Philadelphia, PA, USA), covering publications available up to June 2026. Search terms included combinations of “MASLD”, “MASH”, “NAFLD”, “cardio-oncology”, “cardiotoxicity”, “cancer therapy-related cardiovascular toxicity”, “nutrition”, “diet”, “Mediterranean diet”, “GLP-1 receptor agonists”, “SGLT2 inhibitors”, “resmetirom”, “oxidative stress”, “mitochondrial dysfunction”, and related terms, combined using Boolean operators.

Priority was given to international clinical guidelines, scientific statements, systematic reviews, meta-analyses, randomised controlled trials and large observational studies published in English. Mechanistic and preclinical studies were also considered when necessary to substantiate the biological rationale underlying the proposed MASLD–Cardio-Oncology framework. Additional relevant publications were identified through manual screening of the reference lists of selected articles.

Because this was a narrative rather than a systematic review, the search was intended to support an evidence-based translational overview and not to provide an exhaustive or quantitatively pooled synthesis of the literature. Evidence was critically interpreted according to study design, methodological quality and clinical relevance.

To facilitate interpretation of the available literature, the principal concepts discussed throughout the review were classified according to the current strength and nature of the supporting evidence (Table 1). Particular caution was applied when extrapolating findings from general MASLD populations, experimental models or non-oncological cohorts to patients receiving anticancer therapies. Accordingly, associations between MASLD and CTR-CVT were regarded as hypothesis-generating unless supported by direct clinical evidence.

## 3. MASLD/MASH: Definition, Epidemiology, and Phenotypes

### 3.1. From NAFLD to MASLD: Cardiometabolic Criteria as Diagnostic Gate and Prognostic Markers

The 2023 multinational Delphi consensus redefined the classification of fatty liver disease by introducing steatotic liver disease (SLD) as the overarching category, encompassing metabolic dysfunction-associated steatotic liver disease (MASLD), MASLD with increased alcohol intake (MetALD), SLD due to specific aetiologies and cryptogenic SLD [1]. Unlike the former NAFLD definition, which relied on the exclusion of competing causes, MASLD adopts a positive diagnostic approach, requiring evidence of hepatic steatosis together with at least one cardiometabolic risk factor [1].

The five cardiometabolic criteria, including overweight or obesity, type 2 diabetes mellitus (T2DM) or prediabetes, hypertension, hypertriglyceridaemia and reduced HDL cholesterol, closely overlap with the components of metabolic syndrome, yet their purpose differs substantially [1,30]. Rather than identifying metabolic syndrome itself, they establish the metabolic context in which hepatic steatosis develops and progresses, and should therefore be regarded not only as diagnostic requirements but also as indicators of disease severity and prognosis [31]. An increasing cardiometabolic burden is consistently associated with a greater likelihood of steatohepatitis, clinically significant fibrosis, and adverse hepatic and cardiovascular outcomes [32,33,34,35]. In the 2017–2018 NHANES cohort, each additional metabolic syndrome component approximately doubled the odds of at-risk NASH, with elevated glucose or T2DM, increased waist circumference and low HDL cholesterol emerging as independent risk factors [33]. Likewise, in a large US clinical laboratory database, patients with NASH and multiple metabolic comorbidities had a significantly higher risk of advanced fibrosis [34].

Beyond the cumulative burden of metabolic abnormalities, their individual contributions also appear clinically relevant. Among the individual criteria, glucose dysregulation consistently emerges as the dominant driver of fibrosis risk. In a population-based multicohort study of MASLD, impaired fasting glucose and T2DM conferred an approximately 2- to 4-fold higher risk of hepatic fibrosis and liver-related events compared with normoglycaemic individuals [35], while longitudinal data from the NASH Clinical Research Network showed that worsening glycaemic control and body weight tracked with progression of liver stiffness, and improvements in these parameters with its reduction [36]. Collectively, these findings support a dynamic view of MASLD severity, determined not only by the presence of steatosis but also by the cumulative cardiometabolic burden accompanying the disease. Consistent with this perspective, current European guidelines recommend the systematic non-invasive assessment of liver fibrosis in high-risk individuals, particularly those with T2DM, obesity with additional cardiometabolic risk factors, or persistently abnormal liver enzymes [2].

Within the MASLD spectrum, metabolic dysfunction-associated steatohepatitis (MASH) represents the progressive inflammatory phenotype characterised by steatosis, hepatocellular ballooning and lobular inflammation. Although MASH identifies a biologically active disease state, long-term prognosis is primarily driven by the presence and severity of liver fibrosis; the early identification of clinically significant fibrosis therefore remains the cornerstone of risk stratification, surveillance and therapeutic decision-making [1,2,37].

### 3.2. Global Epidemiology, Disparities and Systemic Burden

The global burden of MASLD has increased dramatically over the past two decades, in parallel with the worldwide epidemics of obesity, T2DM and metabolic syndrome, and MASH, and is now recognised as a leading driver of cirrhosis, hepatocellular carcinoma (HCC) and liver transplantation across many regions of the world [4,5]. Recent estimates indicate a prevalence of approximately 30.4% among adults in the European Union and the United Kingdom [5], with projections exceeding 55% by 2050 [4]; within this expanding population, MASH affects an estimated 5% of the global population [6].

The epidemiology of MASLD shows substantial geographic heterogeneity, reflecting differences in lifestyle, socioeconomic factors, ethnicity and genetic susceptibility. The highest prevalence rates have been reported in the Middle East and Latin America, where the coexistence of obesity, T2DM and population-specific genetic susceptibility may contribute to accelerated disease progression and to increased risks of advanced fibrosis and HCC [38,39]. Several African countries are undergoing a rapid epidemiological transition, with rising obesity and T2DM superimposed on the persistent burden of chronic viral hepatitis and HIV infection, generating unique phenotypes of steatotic liver disease with potentially accelerated fibrogenesis [38]. Italian epidemiological data are consistent with global trends, with particularly high prevalence among individuals with obesity, metabolic syndrome and T2DM [40].

Beyond the classical components of metabolic syndrome, comorbid conditions such as obstructive sleep apnoea (OSA) have been consistently associated with more severe steatohepatitis, advanced fibrosis and an increased risk of long-term hepatic complications, plausibly through chronic intermittent hypoxia, oxidative stress and systemic inflammation [41]. In contrast, although polycystic ovary syndrome (PCOS) is strongly associated with insulin resistance and hepatic steatosis, current evidence linking PCOS to advanced fibrosis remains less consistent [42].

Sex- and gender-related differences further shape the epidemiology of MASLD. The prevalence is higher in men than in premenopausal women, but the gap narrows substantially after menopause, when the decline in circulating oestrogens is accompanied by a marked increase in hepatic steatosis, MASH and advanced fibrosis [43]. Sex-related differences also extend to disease progression and outcomes: men tend to exhibit more rapid fibrosis progression and a higher incidence of HCC, whereas postmenopausal women experience greater cardiovascular mortality. Pregnancy-related factors, including gestational diabetes and maternal high-fat dietary patterns, may further contribute to sex-specific transgenerational transmission of metabolic risk, reinforcing the concept that MASLD develops within a life-course framework rather than as an isolated adult disease [44].

### 3.3. Phenotyping: Beyond a Single Entity

Beyond its global and demographic heterogeneity, MASLD encompasses a heterogeneous spectrum of clinical phenotypes that differ in metabolic profile, genetic susceptibility, disease progression and extrahepatic complications. Although excessive hepatic triglyceride accumulation represents the defining feature of the disease, the clinical course varies considerably among individuals, reflecting the complex interplay between metabolic dysfunction, environmental factors and genetic background [45].

Approximately 7–20% of affected individuals are lean, with prevalence varying across ethnic groups partly because of differences in body fat distribution and ethnicity-specific body mass index (BMI) thresholds. Although lean patients may share core pathogenic mechanisms of MASH with non-lean individuals, important differences have been described in disease progression, associated comorbidities, and diagnostic and therapeutic approaches [45]. Despite normal or only mildly increased BMI, these patients frequently exhibit visceral adiposity, insulin resistance and other metabolic abnormalities, suggesting that adipose tissue dysfunction, rather than body weight per se, drives disease development.

Increasing evidence indicates that lean MASLD should not be considered a benign phenotype. A large retrospective cohort based on the multinational TriNetX federated healthcare database comparing lean and non-lean MASLD reported significantly higher risks of new-onset heart failure, composite cardiovascular events, cerebrovascular events and all-cause mortality in the lean subgroup over a 7-year follow-up [46]. Consistent findings emerged from a nationwide United States (US) Veterans Health Administration cohort of patients with MASLD-related cirrhosis, in which the lean phenotype was associated with higher cardiovascular and all-cause mortality despite comparable rates of major adverse cardiovascular events [47]. Beyond these mortality signals, lean MASLD also appears to confer an adverse cardiometabolic risk profile: in a multicentre study including patients with T2DM from 16 centres in China, lean individuals exhibited a higher prevalence of hypertension and established cardiovascular disease than their non-lean counterparts, despite lower hepatic steatosis and fibrosis burden on transient elastography [48]. Taken together, these observations support the notion that metabolic dysfunction, rather than obesity per se, is the principal determinant of adverse cardiovascular outcomes in MASLD.

Body weight alone also appears to be an imperfect surrogate of liver disease severity. A multicentre biopsy-based analysis of adults enrolled in prospective studies across nine participating US NASH Clinical Research Network centres showed that histological severity was broadly comparable between normal-weight and overweight individuals, whereas a marked increase in steatohepatitis and fibrosis was observed once BMI reached the obesity range (≥30 kg/m^2^) [49]. This threshold is consistent with the established role of obesity in MASLD progression but challenges the assumption that overweight individuals represent an intermediate-risk category between lean and obese patients; notably, approximately one-quarter of normal-weight patients already exhibited advanced fibrosis, indicating that clinically significant liver disease may occur even in the absence of overt obesity.

Genetic susceptibility represents a further major determinant of phenotypic heterogeneity. Among the identified variants, the PNPLA3 rs738409 (I148M) polymorphism remains one of the strongest inherited risk factors for hepatic steatosis, steatohepatitis, advanced fibrosis and cirrhosis [50]. Notably, the risk allele appears particularly enriched among non-obese individuals with MASLD and may contribute to fibrosis progression independently of BMI, further supporting the concept that genetic background modifies disease expression beyond traditional metabolic risk factors [49,50].

Collectively, the observations summarised in this section indicate that MASLD should not be regarded as a homogeneous liver disorder but rather as a systemic cardiometabolic disease whose clinical expression is shaped by geographic, genetic, metabolic and sex-specific factors. This perspective has important implications for risk stratification, preventive strategies and the development of personalised therapeutic approaches, and provides the conceptual basis for the central thesis of this review: the systemic burden of MASLD extends well beyond the liver, contributing to cardiovascular disease, cancer susceptibility and, potentially, increased vulnerability to cancer therapy-related cardiovascular toxicity. Within this framework, distinct MASLD phenotypes may differ in their susceptibility to such toxicity and may therefore warrant individualised preventive and nutritional strategies.

## 4. MASLD and the Cardiovascular Axis

### 4.1. MASLD as Cardiovascular Risk-Enhancing Condition

Cardiovascular disease represents the leading cause of death in patients with MASLD, exceeding liver-related mortality across all disease stages [12,19]. Large meta-analyses have consistently shown that MASLD is associated with increased cardiovascular mortality, incident cardiovascular disease and all-cause mortality, independently of traditional risk factors, and that cardiovascular risk increases progressively with worsening liver histology, indicating that fibrosis stage, rather than steatosis alone, is the principal determinant of adverse outcome [14].

The relationship between MASLD and atherosclerotic cardiovascular disease is supported by both epidemiological and imaging evidence. MASLD has been associated with a 26% higher risk of incident myocardial infarction [51], together with a higher prevalence of coronary artery calcification, epicardial adipose tissue expansion, obstructive coronary artery disease and high-risk plaque features [52,53,54], suggesting that MASLD contributes not only to accelerated atherosclerosis but also to plaque vulnerability and adverse coronary remodelling.

Heart failure with preserved ejection fraction (HFpEF) is among the most clinically relevant cardiovascular outcomes. Several large cohort studies and meta-analyses have shown that MASLD is associated with a significantly increased risk of incident HF, particularly HFpEF, even after adjustment for conventional cardiometabolic risk factors [17,55,56,57,58,59]. This risk appears to rise in parallel with the severity of hepatic disease and the cumulative burden of metabolic abnormalities, supporting the concept that MASLD contributes to myocardial dysfunction through mechanisms extending beyond shared cardiovascular risk factors.

Similarly, growing evidence links MASLD to atrial remodelling and atrial fibrillation (AF) [16]. Meta-analytic data indicate a significantly increased incidence of AF in individuals with MASLD, while markers of advanced liver fibrosis such as elevated FIB-4 scores have been associated with incident arrhythmias and AF recurrence [60], plausibly through an inflammatory signature centred on CXCL10 [61]; MASLD has also been shown to predict AF recurrence after catheter ablation [62]. These observations support the hypothesis that chronic metabolic inflammation, myocardial fibrosis and altered atrial structure contribute to the arrhythmogenic substrate associated with MASLD.

More recently, valvular heart disease has also been investigated within this spectrum: although the evidence remains less consistent than for coronary artery disease or heart failure, several studies have reported associations between MASLD and aortic valve sclerosis, aortic valve calcification and mitral annular calcification, suggesting that systemic metabolic dysfunction may also contribute to valvular degeneration [63,64,65,66,67].

Across all cardiovascular outcomes, hepatic fibrosis refines risk more consistently than steatosis alone: an advanced fibrosis profile has been associated with an approximately 4-fold higher cardiovascular risk, and the coexistence of MASLD with four or more cardiometabolic risk factors further amplified this risk [68]. In the post hoc analysis of the CLEAR Outcomes trial, each one-unit increase in FIB-4 was associated with a higher incidence of the four-component major adverse cardiovascular event composite (MACE-4: comprising cardiovascular death, non-fatal myocardial infarction, non-fatal stroke or coronary revascularisation) [69]. Liver fibrosis assessment is therefore increasingly regarded as a useful tool for cardiovascular risk refinement, although its incremental predictive value remains to be prospectively validated [2,9].

**Figure 1:**
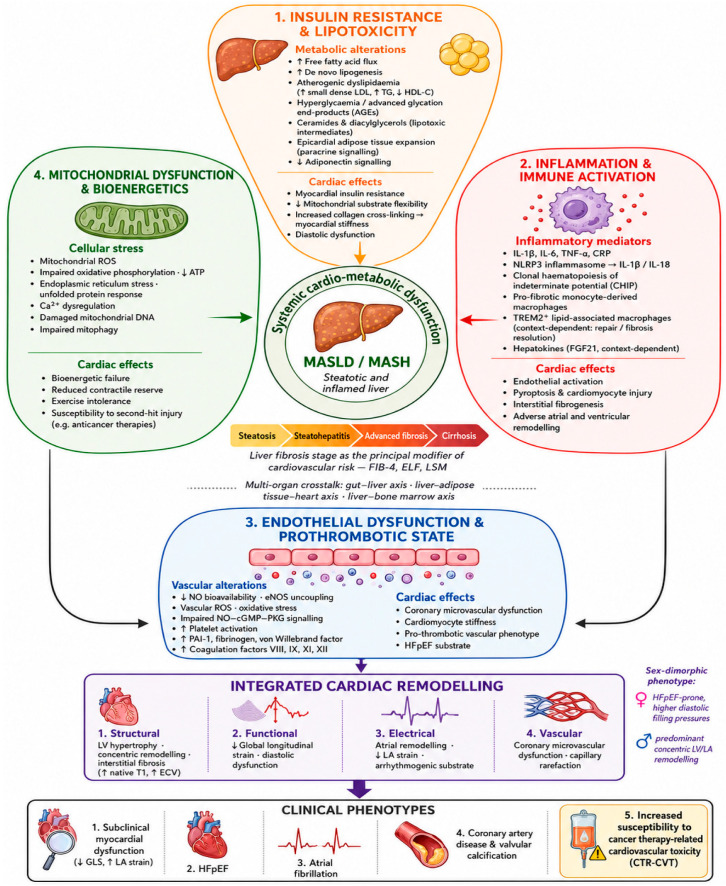
The MASLD-cardiovascular axis: From systemic metabolic dysfunction to integrated cardiac remodelling and increased susceptibility to cancer therapy-related cardiovascular toxicity. *Abbreviations*: AGEs, advanced glycation end-products; ATP, adenosine triphosphate; CHIP, clonal haematopoiesis of indeterminate potential; CRP, C-reactive protein; CTR-CVT, cancer therapy-related cardiovascular toxicity; DAG, diacylglycerol; ECV, extracellular volume; eNOS, endothelial nitric oxide synthase; ER, endoplasmic reticulum; FGF21, fibroblast growth factor 21; FIB-4, fibrosis-4 index; GLS, global longitudinal strain; HDL-C, high-density lipoprotein cholesterol; HFpEF, heart failure with preserved ejection fraction; IL, interleukin; LA, left atrial; LDL, low-density lipoprotein; LSM, liver stiffness measurement; LV, left ventricular; MASH, metabolic dysfunction-associated steatohepatitis; MASLD, metabolic dysfunction-associated steatotic liver disease; NLRP3, NOD-like receptor family pyrin domain-containing 3; NO, nitric oxide; PAI-1, plasminogen activator inhibitor-1; PKG, protein kinase G; ROS, reactive oxygen species; TG, triglycerides; TNF, tumour necrosis factor; TREM2, triggering receptor expressed on myeloid cells 2. *Symbols*: ↑, increased; ↓, decreased.

Collectively, these observations support the concept of MASLD as a systemic cardiometabolic disorder characterised by progressive cardiovascular involvement rather than an isolated hepatic condition, and provide the clinical rationale for examining the biological mechanisms linking MASLD to myocardial injury and its potential relationship with susceptibility to cancer therapy-related cardiovascular toxicity (Figure 1).

### 4.2. Shared Pathophysiological Mechanisms

The association between MASLD and cardiovascular disease is sustained by a complex network of metabolic, inflammatory and vascular interactions involving the liver, adipose tissue, immune system and myocardium. Mechanistic and translational studies suggest that these pathways may converge to promote endothelial dysfunction, myocardial remodelling and progressive cardiovascular injury [9,70,71,72], and many of the same pathways are implicated in cancer therapy-related cardiovascular toxicity, providing the biological rationale for the MASLD–Cardio-Oncology Triangle proposed in this review (Figure 1).

#### 4.2.1. Insulin Resistance and Lipotoxicity

Insulin resistance (IR) is a central driver of MASLD: impaired postprandial anti-lipolytic action of insulin increases free fatty acid (FFA) delivery to the liver, where, once β-oxidation and VLDL secretion are exceeded, FFAs are re-esterified into triglycerides, while hyperinsulinaemia and glucose excess further stimulate de novo lipogenesis [73]. The result is the typical atherogenic dyslipidaemia of MASLD, characterised by hypertriglyceridaemia, increased small, dense LDL and reduced HDL cholesterol [70,71]. Chronic hyperglycaemia and advanced glycation end-products promote endothelial oxidative stress, vascular inflammation and increased myocardial collagen cross-linking, contributing to myocardial stiffness and impaired diastolic compliance [74,75]. In parallel, visceral adipose tissue with IR releases pro-inflammatory adipokines and reduces adiponectin signalling, sustaining a self-perpetuating loop of steatosis, dyslipidaemia, endothelial dysfunction and cardiovascular damage [70,71,72]. Ectopic lipid deposition in skeletal muscle and myocardium promotes local IR, with toxic intermediates (diacylglycerols, ceramides) impairing mitochondrial substrate flexibility and diastolic function [71,72,76], while expanded epicardial adipose tissue (EAT) contributes paracrine pro-inflammatory and pro-fibrotic signals to the underlying myocardium and coronary vasculature [70,71,72,76].

#### 4.2.2. Systemic Inflammation and Immune Activation

Progression from steatosis to MASH is accompanied by chronic low-grade inflammation characterised by elevated circulating concentrations of interleukin (IL)-1β, IL-6, tumour necrosis factor (TNF)-α and C-reactive protein, with the liver amplifying systemic immune activation towards the vascular wall, bone marrow and myocardium [70,71,72]. The NOD-like receptor family pyrin domain-containing 3 (NLRP3) inflammasome appears as a central mediator: saturated fatty acids, cholesterol crystals, ceramides, mitochondrial reactive oxygen species and danger-associated molecular patterns trigger caspase-1-dependent maturation of IL-1β and IL-18, promoting hepatocyte injury, pyroptosis and fibrogenesis [71,72]; elated inflammatory and nitrosative-stress pathways are also implicated in atherosclerosis and experimental HFpEF [72,77].

The haematopoietic compartment may further contribute to this shared inflammatory basis [78]. Clonal haematopoiesis of indeterminate potential (CHIP), particularly involving TET2 mutations, has been associated with adverse cardiac remodelling [79,80] and with NLRP3-dependent steatohepatitis [78,81], while monocyte-derived macrophages may sustain inflammation and fibrosis [71,72,78,82]. Conversely, TREM2^+^ lipid-associated macrophages appear to support tissue repair and fibrosis resolution during MASH regression [83] and may be protective in experimental HFpEF-like remodelling [84].

The steatotic liver also releases hepatokines with heterogeneous, context-dependent cardiovascular effects: FGF21, elevated in both MASH and HFpEF, is generally regarded as cardiometabolically favourable and supports the rationale for FGF21 analogues, although long-term cardiovascular implications remain to be clarified [85].

#### 4.2.3. Endothelial Dysfunction, Oxidative Stress and Prothrombotic State

MASLD has been linked to impaired endothelium-dependent vasodilation and reduced nitric oxide (NO) bioavailability [70,71,72]. Excessive oxidative stress promotes endothelial activation and accelerates atherosclerotic plaque formation [70,71,72,82]; within the coronary microcirculation, chronic metabolic inflammation impairs the NO-cGMP-protein kinase G signalling pathway, favouring cardiomyocyte stiffness, concentric remodelling and diastolic dysfunction, which are hallmarks of HFpEF [77,82].

MASLD is also characterised by a prothrombotic and hypofibrinolytic milieu, with increased platelet activation, elevated plasminogen activator inhibitor-1, fibrinogen, von Willebrand factor and higher activity of coagulation factors VIII, IX, XI and XII [70,71,72,86,87,88]. These abnormalities may contribute not only to thrombotic risk but also to persistent vascular inflammation and adverse myocardial remodelling, reinforcing the systemic cardiovascular burden of advanced metabolic liver disease [86,87,88]. Notably, experimental evidence suggests that the liver-derived coagulation factor XI may also exert paradoxical cardioprotective effects [89], highlighting the complexity of liver-heart crosstalk in chronic metabolic disease.

#### 4.2.4. Mitochondrial Dysfunction and Bioenergetic Failure

Excess FFAs and lipid intermediates may overwhelm cardiomyocyte mitochondria, increasing reactive oxygen species, impairing oxidative phosphorylation and reducing ATP availability; the resulting bioenergetic failure contributes to exercise intolerance and diastolic dysfunction in HFpEF [71,72,76,77,78], and analogous mitochondrial stress occurs in MASLD/MASH [21,22,71,72]. Endoplasmic reticulum stress provides a further point of convergence, disrupting protein folding, intracellular calcium homeostasis and mitochondrial function. Collectively, mitochondrial dysfunction, oxidative stress and impaired cellular bioenergetics integrate the metabolic, inflammatory and vascular abnormalities of MASLD into a unified pathophysiological framework linking hepatic steatofibrosis with myocardial remodelling [21,72,76,77,78].

Several of these pathways also participate in tumour biology and in cardiovascular injury associated with anticancer therapies. Their oncological implications and the hypothesis that MASLD may modify susceptibility to CTR-CVT are considered separately in Section 5.2 and Section 6.3, respectively. This potential interaction remains supported predominantly by mechanistic and indirect clinical evidence rather than by prospective demonstrations of causality.

### 4.3. From Subclinical Cardiac Remodelling to HFpEF and Atrial Fibrillation

The metabolic, inflammatory and vascular mechanisms outlined above ultimately converge towards structural and functional cardiac remodelling.

#### 4.3.1. Left Ventricular Remodelling and Subclinical Myocardial Dysfunction

Subclinical cardiac dysfunction is frequently detectable before the onset of clinical cardiovascular disease. Echocardiographic studies have associated MASLD with altered left ventricular (LV) geometry, dominated by concentric remodelling and concentric hypertrophy even in the absence of arterial hypertension [58], resembling the structural phenotype frequently observed in HFpEF [90]. Speckle-tracking echocardiography has further demonstrated reductions in global longitudinal strain (GLS) despite preserved LV ejection fraction, indicating that impairment of myocardial mechanics precedes overt systolic dysfunction [91]. These findings support the use of myocardial deformation imaging as a sensitive tool for identifying early cardiovascular involvement in patients with MASLD.

#### 4.3.2. Sex-Related Differences

Cardiac remodelling in MASLD appears to differ by sex: men exhibit more pronounced LV and left atrial concentric remodelling with preserved right ventricular function, whereas women show preserved LV systolic function alongside higher diastolic filling pressures and a greater inflammatory burden [92]. This pattern is biologically coherent with the protective effect of endogenous oestrogen and the female predominance of HFpEF [90] and suggests that screening pathways may benefit from sex-stratified interpretation, an aspect still overlooked in current cardiometabolic guidelines [92].

#### 4.3.3. Interstitial Myocardial Fibrosis

Interstitial fibrosis, quantified by cardiac magnetic resonance native T1 and extracellular volume (ECV), is a major determinant of myocardial stiffness, diastolic dysfunction and arrhythmogenic vulnerability; ECV-quantified fibrosis affects more than 41% of HFpEF patients and carries independent prognostic value [90]. Consistently, in biopsy-confirmed MASLD cohorts, advanced hepatic fibrosis has been associated with higher myocardial ECV, supporting the concept of a shared liver–heart fibrotic axis in which fibrotic burden, rather than steatosis alone, may identify patients with greater cardiovascular vulnerability [93].

#### 4.3.4. Atrial Remodelling and Arrhythmogenesis

Left atrial (LA) dilation and impaired LA strain, driven by elevated LV filling pressures, diastolic dysfunction and chronic volume overload, are common in MASLD and represent the haemodynamic substrate for AF [91,94,95]. Higher EAT volume and an altered EAT phenotype correlate with MASLD severity and with both AF incidence and recurrence after catheter ablation, supporting a possible liver–adipose tissue–heart axis linking MASLD pathobiology to arrhythmogenesis [60,62,95,96].

Integrating these observations, MASLD-associated cardiovascular involvement may be best conceptualised as a continuum beginning with subclinical alterations in myocardial mechanics (reduced GLS, increased LA strain) and adverse LV geometry, progressing through interstitial fibrosis (elevated native T1 and ECV) and culminating in overt HFpEF and AF, with prognosis graded by hepatic and myocardial fibrotic burden and sex-dimorphic phenotypic expression. Although causal relationships remain to be fully established, this continuum provides a biologically plausible substrate that may increase myocardial susceptibility to additional insults, including cancer therapy-related cardiovascular toxicity. Combined assessment of hepatic fibrosis and subclinical cardiac dysfunction therefore warrants prospective evaluation as a complementary strategy for identifying patients at increased cardiovascular risk within the emerging cardio-oncology framework.

## 5. MASLD and Oncological Risk

### 5.1. HCC on MASH and Extra-Hepatic Cancers

Metabolic dysfunction-associated steatotic liver disease (MASLD) has become one of the fastest-growing causes of hepatocellular carcinoma (HCC) worldwide, in parallel with the global epidemics of obesity and type 2 diabetes [2,97,98,99]. Between 2010 and 2019, MASH represented the fastest-growing aetiology of incident liver cancer globally [100], and epidemiological models estimate that new MASLD-related HCC cases will almost double over the coming decades [97]. Large meta-analyses and population-based studies confirm that MASLD is associated with a substantial excess risk of HCC [19,98], with the magnitude of risk rising sharply with advancing fibrotic burden, particularly in patients with advanced fibrosis or cirrhosis, supporting fibrosis stage as the major determinant of MASLD-related HCC risk [37,101]. A clinically distinctive feature of MASLD-related HCC is the relatively high proportion of non-cirrhotic cases, which are often diagnosed at older age, with larger tumours and outside established surveillance programmes [98,102,103]. Overall survival does not differ between patients with MASLD-related HCC and those with HCC of other aetiologies, whereas disease-free survival appears to be longer in patients with MASLD-related HCC [104]. Current EASL and AASLD recommendations restrict HCC surveillance to patients with advanced fibrosis or cirrhosis [2,105], while risk-stratified approaches are being explored to identify non-cirrhotic MASLD patients who may benefit from earlier screening [102,106,107].

Beyond the liver, MASLD is consistently associated with a moderately increased risk of several extrahepatic malignancies [18,101,108]. A meta-analysis of 18 observational cohort studies including approximately 16.7 million individuals from seven countries (South Korea, China, Japan, Sweden, the United States, Germany and the United Kingdom) reported significant excess risks for gastrointestinal cancers, including gastric, colorectal, pancreatic and biliary tract neoplasms, as well as for cancers of the thyroid, urinary tract, breast and female reproductive organs [108]. Population-based studies further support these observations, reporting particularly strong associations for HCC together with smaller, but significant, increases in colorectal, kidney, bladder and uterine cancers [109].

Importantly, the observed excess oncological risk appears to be driven primarily by the metabolic component of MASLD rather than by steatosis per se, while sex-specific differences may contribute to variability in cancer susceptibility across different populations [108,109,110]. This concept reinforces the view of MASLD as a systemic metabolic disease whose clinical consequences extend well beyond the liver and provides the biological rationale for the mechanistic pathways discussed in the following section. Because these data are predominantly observational, causal attribution remains uncertain.

### 5.2. Mechanisms: Hyperinsulinaemia, IGF-1, Chronic Inflammation, Tumour Microenvironment, Gut Microbiota and Sex Steroids

Many of the biological pathways linking MASLD with cardiovascular disease, including insulin resistance, chronic low-grade inflammation, endothelial dysfunction and mitochondrial impairment, already discussed in Section 4.2, may also contribute to tumour initiation and progression by creating a pro-tumorigenic metabolic and immune microenvironment. Therefore, this section focuses on their specific relevance to carcinogenesis and tumour progression.

#### 5.2.1. Hyperinsulinaemia and IGF-1 Signalling

Hyperinsulinaemia and dysregulated insulin/IGF signalling are hallmark features of MASLD and provide a biologically plausible link between metabolic dysfunction and carcinogenesis. Sustained insulin elevation exerts direct mitogenic and anti-apoptotic effects and indirectly amplifies IGF-1 signalling by reducing IGF-binding proteins, thereby activating the PI3K/Akt/mTOR and RAS/RAF/MAPK pathways, which promote proliferation, inhibit apoptosis and modulate angiogenesis [19,111,112,113]. These mechanisms have been implicated in several malignancies, including breast, endometrial and colorectal cancers [114,115,116]. Notably, circulating IGF-1 concentrations may decline with increasing MASLD severity and fibrosis, highlighting the complex and tissue-specific dysregulation of the GH-IGF-1 axis rather than its uniform systemic activation [117].

#### 5.2.2. Chronic Inflammation and Tumour Microenvironment

Building upon the inflammatory pathways described in Section 4.2, chronic immune activation in MASLD contributes to the establishment of a tumour-permissive microenvironment that promotes DNA damage, genomic instability and carcinogenesis [118,119]. MASLD-related HCC also displays a distinct tumour microenvironment, characterised by exhausted CD8^+^ T cells, TREM2^+^ lipid-associated macrophages and impaired NKT cell function. Experimental and translational evidence suggests that these alterations may facilitate tumour progression and potentially modify responses to immune checkpoint inhibitors; however, their clinical relevance remains incompletely defined [120,121,122,123,124,125].

#### 5.2.3. Gut–Liver Axis and Microbial Dysbiosis

Experimental and translational evidence implicates the gut–liver axis through intestinal barrier disruption, portal translocation of lipopolysaccharide and other microbial-associated molecular patterns, and alterations in microbial metabolite and bile acid composition [126,127]. Secondary bile acids, particularly deoxycholic acid, may promote oxidative DNA damage and cellular senescence, generating a senescence-associated secretory phenotype that favours hepatic inflammation and tumourigenesis, while dysregulated FXR/TGR5 signalling may further disrupt metabolic and immune homeostasis [128]. Microbial metabolites, including short-chain fatty acids (SCFAs) and tryptophan derivatives, exert context-dependent immunometabolic effects: in MASLD-related HCC, an SCFA-enriched microbial profile has been associated with regulatory T-cell expansion and attenuation of cytotoxic CD8^+^ T-cell responses [126,127,129]. These findings remain predominantly mechanistic or associative and have yet to establish a causal role for specific microbiota profiles in cancer development or treatment response.

#### 5.2.4. Sex Hormones and Endocrine Regulation

MASLD modulates sex steroid bioavailability through reduced hepatic synthesis of sex hormone-binding globulin, increasing free fractions of oestrogens and androgens and potentially contributing to endometrial, breast and prostate carcinogenesis, with implications for the sex-specific epidemiology of MASLD-related cancers [108,130]. These endocrine alterations may also partly explain the sex-related differences observed in the epidemiology of MASLD-associated cancers.

Overall, current mechanistic models propose that the increased cancer risk associated with MASLD may reflect the interaction of metabolic dysfunction, chronic inflammation, immune remodelling, gut microbial dysbiosis and endocrine disturbances rather than hepatic steatosis alone. Many of the pathways involved are shared with those discussed in Section 4.2, reinforcing the concept of a common metabolic-inflammatory substrate linking liver disease, cardiovascular disease and cancer. However, although this mechanistic overlap provides a strong biological rationale for the proposed MASLD–Cardio-Oncology framework, its direct implications remain hypothetical and require prospective validation.

## 6. Cardiotoxicity of Oncological Therapies and the Modulatory Role of MASLD

### 6.1. ESC 2022 Framework and HFA-ICOS Risk Stratification

The growing population of long-term cancer survivors has made cancer therapy-related cardiovascular toxicity (CTR-CVT) a major determinant of morbidity, mortality and quality of life, driving the development of cardio-oncology as a multidisciplinary discipline dedicated to cardiovascular prevention throughout the cancer continuum. The 2022 European Society of Cardiology (ESC) Guidelines on Cardio-Oncology established a comprehensive framework for cardiovascular care across the cancer pathway, emphasising that treatment-related risk depends not only on drug toxicity but also on the patient’s baseline cardiovascular profile [25].

To operationalise this approach, the ESC Guidelines endorse the Heart Failure Association-International Cardio-Oncology Society (HFA-ICOS) risk assessment tools, which provide therapy-specific risk stratification models for patients scheduled to receive potentially cardiotoxic treatments, including anthracyclines, HER2-targeted agents, VEGF inhibitors, BCR-ABL tyrosine kinase inhibitors, proteasome inhibitors, RAF/MEK inhibitors and androgen-deprivation therapies [24]. The algorithm incorporates a broad spectrum of variables, such as demographic characteristics, cardiovascular history, conventional cardiovascular risk factors, previous cardiotoxic exposure, biomarkers and cardiac imaging, to classify patients into low-, moderate-, high- or very-high-risk categories [24]. Importantly, the weighting assigned to each variable differs according to the specific anticancer therapy under consideration, reflecting the heterogeneous mechanisms of CTR-CVT [24]. This individualised approach represents a substantial advancement compared with previous one-size-fits-all surveillance strategies and is currently recommended as the standard method for baseline cardiovascular risk assessment in oncology patients [25].

Despite these advances, the current HFA-ICOS model remains largely centred on traditional cardiovascular risk factors and established cardiovascular disease. Liver-related variables, including hepatic steatosis, fibrosis stage, liver stiffness measurements, or non-invasive fibrosis scores such as FIB-4 and ELF, are not included in current risk algorithms [24,25]. This omission may become increasingly relevant given the growing prevalence of MASLD among patients with cancer and the accumulating evidence identifying MASLD as an independent cardiovascular risk enhancer. Indeed, several studies have demonstrated that MASLD, particularly when accompanied by advanced fibrosis, is associated with increased risks of major adverse cardiovascular events, HF, AF, endothelial dysfunction and cardiovascular mortality [9,14,16]. Consequently, a substantial proportion of contemporary oncology patients may enter treatment with an underlying metabolic-inflammatory and fibrotic substrate that is not adequately captured by existing cardio-oncology risk scores.

Furthermore, recent validation studies have confirmed the clinical utility of HFA-ICOS risk stratification while simultaneously highlighting its limitations [26,131,132]. Although the score performs reasonably well in identifying patients at increased risk of treatment-related cardiotoxicity, predictive accuracy remains only moderate in some clinical settings, particularly for subclinical cardiac dysfunction and across different tumour types. Emerging evidence suggests that integrating additional markers of metabolic and cardiovascular vulnerability, including body composition, vascular function and imaging-derived myosteatosis, may improve risk discrimination beyond conventional cardiovascular variables [133,134].

Within this context, MASLD has emerged as a potential modifier of baseline cardio-oncology risk; however, its incremental predictive value beyond established HFA-ICOS variables has yet to be prospectively demonstrated. The pathophysiological overlap between MASLD and CTR-CVT, including mitochondrial dysfunction, oxidative stress, chronic low-grade inflammation, endothelial impairment and pro-fibrotic signalling, provides a biological rationale for integrating liver phenotyping into future cardio-oncology risk models. Such an approach would align with the emerging concept of cardiovascular–liver–metabolic health and may improve risk stratification in an increasingly metabolically complex oncology population [9]. Accordingly, MASLD should be viewed not merely as a metabolic comorbidity but as a potential modifier of baseline cardio-oncology risk, deserving prospective evaluation in future refinements of HFA-ICOS-based stratification models.

### 6.2. Therapy-Related Cardiovascular Injury Pathways

Relevant to MASLD, CTR-CVT encompasses a heterogeneous spectrum of myocardial, vascular and immune-mediated complications that differ according to the mechanism of action of anticancer agents, cumulative drug exposure and patient-related susceptibility. The 2022 ESC Cardio-Oncology Guidelines classify CTR-CVT according to clinical phenotype, including asymptomatic cancer therapy-related cardiac dysfunction, symptomatic HF, myocarditis, vascular toxicity, hypertension, ischaemia, arrhythmias, thromboembolism and pulmonary hypertension [25]. Although individual anticancer classes activate partly distinct mechanisms, these frequently converge on a limited number of biological pathways, including mitochondrial dysfunction, oxidative stress, endothelial injury, inflammation and maladaptive myocardial remodelling [25,135]. These pathways overlap with several biological abnormalities described in MASLD, but their potential interaction with the MASLD phenotype remains supported predominantly by mechanistic and indirect evidence. Representative agents, clinical phenotypes, principal mechanisms and pathways potentially shared with MASLD are summarised in Table 2. Four mechanistic axes are particularly relevant to a MASLD substrate.

First, mitochondrial injury, oxidative stress and disrupted iron handling dominate anthracycline-induced cardiomyopathy through topoisomerase IIβ-mediated DNA double-strand breaks, the suppression of mitochondrial biogenesis, excessive ROS generation, iron overload, ferroptosis and impaired calcium handling [135,136,137].

Second, disrupted cardiomyocyte stress-adaptation and proteostasis underlie HER2-targeted-induced reversible left-ventricular dysfunction, through the inhibition of neuregulin-1/ERBB2-ERBB4 survival signalling [136,137,138], as well as the heart failure, hypertension and vascular events observed with proteasome inhibitors, particularly carfilzomib, which impair endoplasmic-reticulum proteostasis and mitochondrial function.

Third, endothelial and microvascular toxicity constitutes the shared mechanistic core of fluoropyrimidine-associated coronary vasospasm and ischaemia [139,140] and of the hypertension, left ventricular dysfunction and arterial thromboembolism observed with vascular endothelial growth factor-pathway inhibitors and multi-target tyrosine kinase inhibitors [141,142]. This category also encompasses the prothrombotic and vascular phenotype of immunomodulatory drugs and the arterial occlusive or pulmonary vascular events associated with BCR-ABL tyrosine kinase inhibitors such as nilotinib, ponatinib and dasatinib [143,144]. All these agents converge on impaired nitric oxide signalling, endothelial injury and vascular remodelling.

Fourth, immune-mediated myocardial injury represents the defining feature of immune checkpoint inhibitor-associated myocarditis, driven by the loss of peripheral immune tolerance, expansion of autoreactive T-cell clones and myocardial immune infiltration [25,145,146].

**Table 2:** Major anticancer therapies, cardiotoxicity phenotypes and mechanistic overlap with MASLD-related pathways.

Drug Class	Representative Agents	Predominant Cardiotoxicity	Principal Mechanisms	Pathways Shared with MASLD
**Anthracyclines**	Doxorubicin, epirubicin, daunorubicin	HF, LV systolic dysfunction; late dilated cardiomyopathy	TOP2β-mediated DNA double-strand breaks; mitochondrial ROS; impaired mitochondrial biogenesis; cardiolipin binding; iron overload; ferroptosis [135,147,148]	Mitochondrial dysfunction; oxidative stress; impaired fatty-acid β-oxidation; lipotoxicity; hepatic and myocardial iron handling abnormalities [70,71,72,78]
**HER2-targeted agents**	Trastuzumab, pertuzumab, T-DM1	Reversible “type II” LV dysfunction, HF	Inhibition of neuregulin-1/ERBB2-ERBB4 signalling; reduced cardiomyocyte stress adaptation and repair [136,137,138]	Lipotoxicity; reduced cardiomyocyte metabolic flexibility; ↓ myocardial reserve in metabolic/insulin-resistant substrate; loss of stress-induced survival signalling [70,72]
**Fluoropyrimidines**	5-Fluorouracil, capecitabine	Coronary vasospasm, ischaemia, ACS, arrhythmias	Endothelial dysfunction; impaired NO signalling; ↑ endothelin-1; microvascular dysfunction; oxidative and inflammatory injury [139,140]	Endothelial dysfunction; ↓ NO bioavailability; endothelin-1 activation; microvascular rarefaction [70,71]
**VEGF-pathway inhibitors**	Bevacizumab; sunitinib, sorafenib, pazopanib, axitinib, lenvatinib, cabozantinib	Hypertension, LV dysfunction, HF, arterial thromboembolism	VEGF inhibition: ↓ endothelial NO and prostacyclin; ↑ endothelin-1; capillary rarefaction; ↑ systemic vascular resistance [25,141,142]	Endothelial dysfunction; ↓ NO bioavailability; microvascular rarefaction; pro-hypertensive vascular remodelling [70,71]
**Proteasome inhibitors**	Carfilzomib (bortezomib, ixazomib)	HF, hypertension, arrhythmias, ACS, pulmonary hypertension	Proteotoxic stress from misfolded protein accumulation; endothelial dysfunction; ↓ NO; mitochondrial injury; oxidative stress [149]	ER stress; unfolded protein response; mitochondrial dysfunction; chronic low-grade inflammation [70,72]
**IMiDs**	Thalidomide, lenalidomide, pomalidomide	Venous thromboembolism	Cancer-related hypercoagulability + endothelial activation + inflammatory and prothrombotic signalling [150]	Pro-thrombotic milieu; endothelial activation; systemic inflammation [70,71]
**BCR-ABL TKIs**	Nilotinib, ponatinib (arterial); dasatinib (PH/pleural effusion)	Arterial occlusive events (coronary, cerebrovascular, PAD); pulmonary arterial hypertension	Endothelial injury; pro-thrombotic EC phenotype; vascular smooth-muscle dysfunction; metabolic derangement [143,144]	Endothelial dysfunction; insulin resistance and dyslipidaemia (nilotinib); accelerated atherosclerosis [70,71,78]
**Immune checkpoint inhibitors**	Anti-CTLA-4 (ipilimumab); anti-PD-1 (nivolumab, pembrolizumab); anti-PD-L1	Immune-mediated myocarditis (rare but high-fatality); pericarditis; arrhythmias; accelerated atherosclerosis	Loss of peripheral immune tolerance; expansion of autoreactive T-cell clones; myocardial immune infiltration; cytokine release [25,145,146]	Chronic low-grade inflammation; NLRP3 inflammasome activation; immune dysregulation in MASH [70,72]

*Abbreviations*: ACS, acute coronary syndrome; EC, endothelial cell; ER, endoplasmic reticulum; HF, heart failure; IMiDs, immunomodulatory drugs; LV, left ventricular; MASH, metabolic dysfunction-associated steatohepatitis; MASLD, metabolic dysfunction-associated steatotic liver disease; NLRP3, NOD-like receptor family pyrin domain-containing 3; NO, nitric oxide; PAD, peripheral arterial disease; PH, pulmonary hypertension; ROS, reactive oxygen species; T-DM1, trastuzumab emtansine; TKI, tyrosine kinase inhibitor; TOP2β, topoisomerase IIβ; VEGF, vascular endothelial growth factor. *Symbols*: ↑, increased; ↓, decreased.

### 6.3. Why MASLD May Amplify Cardiotoxicity: Shared Substrate, Pharmacokinetics, Sarcopenic Obesity and Causal Uncertainty

Although MASLD is increasingly recognised as a major cardiovascular risk enhancer [9,14,19,70], its potential role as a modifier of CTR-CVT remains insufficiently investigated. As detailed in Section 4.2, MASLD generates a multi-organ network of metabolic, inflammatory, endothelial, mitochondrial and proteostatic perturbations that converge on the myocardium and the vasculature [70,71,72,78]. Many of these same pathways are reactivated and amplified by anticancer therapies (Section 6.2), providing a coherent biological rationale for the hypothesis that MASLD may act as a “second-hit” substrate that lowers the threshold for treatment-induced cardiac and vascular injury (Table 2) [19,25,135,142]; however, it requires prospective clinical validation, because evidence supporting this interaction remains predominantly indirect.

#### 6.3.1. Shared Metabolic and Inflammatory Substrate

The metabolic and inflammatory pathways described in Section 4.2 may define a pre-existing cardiometabolic phenotype characterised by insulin resistance, lipotoxicity, chronic low-grade inflammation and impaired myocardial adaptation. Their potential relevance to CTR-CVT lies not in the presence of a unique MASLD-specific mechanism, but in the possibility that treatment-related injury is superimposed on an already altered metabolic and inflammatory substrate. Several of these pathways are also implicated in the pathogenesis of CTR-CVT: anthracyclines induce excess mitochondrial ROS and oxidative DNA damage, and activate inflammatory and ferroptotic signalling [135,147,148]; fluoropyrimidines promote endothelial dysfunction and vascular inflammation [139,140]; proteasome inhibitors trigger proteotoxic stress, NF-κB activation and endothelial dysfunction [149]; and immune checkpoint inhibitors may facilitate expansion of autoreactive T-cell clones in a myocardium that is already exposed to low-grade immune activation [145,146]. Therefore, rather than representing independent mechanisms, these treatment-related insults may be superimposed on a pre-existing metabolically compromised myocardium, potentially amplifying cardiovascular injury. However, this concept is currently supported mainly by mechanistic and translational evidence, whereas direct clinical confirmation remains limited.

#### 6.3.2. Endothelial Dysfunction and Vascular Vulnerability

Endothelial dysfunction represents a plausible biological bridge between MASLD and CTR-CVT. As described in Section 4.2, MASLD is associated with impaired endothelium-dependent vasodilation, reduced nitric oxide (NO) bioavailability, oxidative stress and a prothrombotic, hypofibrinolytic profile [70,71,72,86,87,88]. Because fluoropyrimidines, VEGF-pathway inhibitors and BCR-ABL TKIs also affect endothelial and microvascular homeostasis, pre-existing vascular impairment could theoretically increase susceptibility to coronary vasospasm, treatment-related hypertension, myocardial ischaemia and microvascular dysfunction [139,140,141,142,143,144].

Consequently, patients with MASLD may enter oncological treatment with pre-existing vascular impairment, potentially increasing susceptibility to coronary vasospasm, treatment-related hypertension, myocardial ischaemia and microvascular dysfunction. Consistent with this view, the severity of hepatic fibrosis correlates with arterial stiffness, high-risk coronary plaque burden, subclinical atherosclerosis and adverse cardiovascular outcomes [14,52,53,68]. Whether pre-existing endothelial dysfunction associated with MASLD independently increases the risk of CTR-CVT has yet to be demonstrated in prospective clinical studies.

#### 6.3.3. Mitochondrial Dysfunction, Proteostatic Stress and Reduced Metabolic Resilience

Mitochondrial dysfunction and endoplasmic-reticulum stress, described in Section 4.2, provide another potential point of interaction with anticancer therapy-related injury. MASLD and MASH are associated with impaired fatty-acid β-oxidation, increased ROS production, reduced metabolic flexibility and activation of the unfolded protein response [21,71,76,77,78]. Anthracyclines and proteasome inhibitors activate overlapping mitochondrial and proteostatic injury pathways, as summarised in Section 6.2 [135,147,148,149]. Mechanistic considerations therefore suggest that reduced metabolic and antioxidant reserve could influence myocardial tolerance to these therapies. Nevertheless, evidence directly demonstrating that a MASLD-associated myocardial phenotype increases anthracycline- or proteasome inhibitor-related cardiotoxicity remains limited.

Observational evidence, derived largely from non-oncological cohorts, has been associated with subclinical myocardial dysfunction, impaired global longitudinal strain, increased left ventricular mass, myocardial fibrosis, heart failure and atrial fibrillation [15,55,59,91,93,94]. Cardio-oncology studies have also shown that body-composition abnormalities, including myosteatosis, may predict anthracycline-related cardiotoxicity and improve HFA-ICOS risk discrimination [131,132,133,134]. Collectively, these findings support the hypothesis that selected metabolic and body-composition phenotypes associated with MASLD may identify reduced myocardial reserve. However, whether hepatic steatosis or fibrosis provides prognostic information beyond these coexisting factors remains uncertain.

#### 6.3.4. Pharmacokinetic Considerations

In addition to shared biological mechanisms, MASLD may influence the pharmacokinetics of several anticancer agents. Steatosis, steatohepatitis and fibrosis alter hepatic blood flow, cytochrome P450 activity, drug transporters and inflammatory pathways regulating drug metabolism [21,70,151,152]. These alterations may modify systemic exposure to anthracyclines, taxanes, tyrosine kinase inhibitors and other agents predominantly cleared by the liver, thereby contributing to inter-individual variability in toxicity. Although direct clinical evidence remains limited, future pharmacokinetic studies stratified according to MASLD phenotype and fibrosis stage are warranted to determine whether liver phenotyping may guide individualised treatment monitoring or dosing strategies.

#### 6.3.5. Sarcopenic Obesity as an Additional Vulnerability Factor

Sarcopenic obesity represents an additional candidate vulnerability factor that may contribute to treatment toxicity within the MASLD phenotype. Many patients with MASLD exhibit a paradoxical phenotype characterised by excess adiposity combined with reduced skeletal muscle mass and impaired muscle function [6,19,153,154]. Sarcopenic obesity has emerged as an important predictor of chemotherapy toxicity, treatment discontinuation, dose reductions and reduced survival across multiple cancer types [155,156], and recent imaging studies have specifically identified myosteatosis as an independent predictor of anthracycline-related cardiotoxicity in lymphoma cohorts [133].

Because conventional chemotherapy dosing is generally based on body surface area rather than on body composition, patients with low lean body mass may receive relatively higher effective drug exposure, increasing susceptibility to toxicity [155,156]. Moreover, skeletal muscle is a major site of glucose disposal, amino acid handling and exercise-induced anti-inflammatory signalling; loss of muscle mass may therefore further exacerbate the insulin resistance, dyslipidaemia and chronic inflammation that already characterise MASLD [6,19,70,153], narrowing both the metabolic and the cardiovascular reserve required to tolerate anticancer therapy.

Overall, the remarkable overlap between MASLD pathophysiology and the mechanisms underlying CTR-CVT supports a unifying conceptual model in which MASLD may identify a systematic state of increased cardiovascular vulnerability rather than a passive comorbidity. However, the available evidence does not establish whether MASLD itself independently amplifies treatment-related toxicity or primarily reflects the cumulative burden of metabolic dysfunction, inflammation, endothelial injury, mitochondrial impairment and adverse body composition. This uncertainty provides the rationale for the explicit consideration of residual confounding, reverse causality and causal inference in the following section.

#### 6.3.6. Residual Confounding, Reverse Causality and Causal Uncertainty

Several important caveats should be acknowledged when interpreting the framework outlined above. Most of the evidence linking MASLD to cardiovascular vulnerability and reduced tolerance of anticancer therapy derives from observational studies and is therefore susceptible to residual confounding. Obesity, visceral adiposity, insulin resistance, type 2 diabetes, sarcopenia and myosteatosis are major determinants of MASLD [8,30,153,154,157] and may also independently increase susceptibility to CTR-CVT [133,155,156]. Accordingly, it remains uncertain whether MASLD exerts an independent causal effect on cardiotoxicity, acts as a mediator or effect modifier of shared cardiometabolic pathways, or primarily represents an integrated marker of a broader metabolic-inflammatory phenotype.

Reverse causality must also be considered. Cancer-related inflammation, reduced physical activity, glucocorticoid exposure, treatment-related metabolic changes, weight fluctuations and muscle loss may induce or worsen insulin resistance, hepatic steatosis, sarcopenia and myosteatosis [155,156,158]. Conversely, chronic cardiac dysfunction, systemic inflammation and haemodynamic or metabolic stress may contribute to hepatic injury, supporting a bidirectional rather than strictly unidirectional cardio–hepatic relationship [78]. MASLD identified during or after anticancer treatment may therefore be, at least in part, a consequence of cancer, its therapies or evolving cardiovascular dysfunction rather than exclusively a pre-existing determinant of treatment-related toxicity.

This distinction has important clinical implications but does not necessarily eliminate the potential value of hepatic phenotyping. Even if MASLD primarily reflects cumulative cardiometabolic vulnerability rather than an independent causal exposure, its assessment may still provide incremental information for identifying patients with reduced cardiovascular reserve and for tailoring surveillance, although this hypothesis requires prospective validation [24,26]. Clarifying the causal contribution of MASLD will require cardio-oncology cohorts incorporating baseline and serial hepatic and body-composition phenotyping, treatment-specific adjudication of CTR-CVT, and analytical approaches capable of distinguishing confounding, mediation and interaction [24,26,131]. Mendelian randomisation studies using carefully selected genetic instruments associated with hepatic steatosis or fibrosis, including variants in PNPLA3, TM6SF2 and HSD17B13, may provide complementary evidence [159], while interventional studies will be needed to determine whether improvement in hepatic phenotype translates into reduced cardiovascular toxicity. At present, MASLD should therefore be regarded as a biologically plausible, candidate integrated substrate and risk marker, rather than an established independent causal determinant of CTR-CVT. Nevertheless, the convergence of metabolic, inflammatory, endothelial and mitochondrial pathways described above supports its prospective evaluation within the MASLD–Cardio-Oncology Triangle.

## 7. Nutrition, Diet, and Lifestyle: From MASLD Prevention to Cardioprotection in Patients with Cancer

### 7.1. Dietary Patterns and MASLD: Evidence and Clinical Applicability

Lifestyle modification remains the cornerstone of MASLD management and is recommended as first-line therapy alongside optimal treatment of cardiometabolic comorbidities, as endorsed by international clinical guidelines including the EASL-EASD-EASO 2024 Clinical Practice Guidelines on MASLD and the AASLD guidance for NAFLD/MASLD [2]. Although weight loss is a major determinant of hepatic improvement, dietary quality and composition may also influence hepatic steatosis, insulin sensitivity and cardiovascular risk [160]. This is particularly relevant in cardio-oncology, where nutritional interventions must balance metabolic improvement with the preservation of nutritional status, skeletal muscle mass and tolerance of anticancer treatment (Table 3). Guideline recommendations support lifestyle intervention for MASLD, whereas evidence for the prevention of CTR-CVT through dietary modification remains emerging.

Among available dietary strategies, the Mediterranean diet (MD) has the most consistent evidence supporting combined hepatic and cardiovascular benefits. It is characterised by a high intake of vegetables, fruit, legumes, whole grains, nuts, extra-virgin olive oil and fish; moderate consumption of dairy products; and limited intake of red and processed meat, refined carbohydrates and added sugars [161,162]. Its potential benefits are mediated through favourable effects on insulin sensitivity, lipid metabolism, oxidative stress, inflammatory signalling and endothelial function. Randomised controlled trials and meta-analyses of interventional data suggest that Mediterranean-style interventions may reduce intrahepatic fat and improve selected metabolic parameters, including insulin sensitivity and liver enzymes, although findings are heterogeneous [163,164,165]. Notably, the MEDINA trial found no significant between-group differences in hepatic or metabolic outcomes when the MD was compared with a low-fat diet, highlighting the importance of energy intake, adherence and the nutritional quality of the comparator diet [164]. Observational evidence has also associated greater MD adherence with a lower risk of incident hepatic steatosis and cardiovascular events in broader cardiometabolic populations, while studies in patients with fatty liver disease have reported lower platelet activation and hepatic collagen deposition [166,167,168,169]. In the context of cardio-oncology, no dedicated randomised trials have yet evaluated the impact of Mediterranean-style dietary patterns on CTR-CVT; the available evidence in cancer survivors derives from observational cohorts and small pilot interventions, and the corresponding recommendations should therefore be regarded as hypothesis-generating extrapolations from the general cardiometabolic setting. The MD should therefore be regarded as a flexible, evidence-supported dietary framework.

The Dietary Approaches to Stop Hypertension (DASH) diet shares several characteristics with the MD, including a high intake of fruit, vegetables, legumes and whole grains and a low intake of sodium, processed meat and refined sugars [170]. Meta-analytic evidence suggests modest reductions in liver enzymes, although data on liver histology and long-term MASLD outcomes remain limited [171]. Because hypertension is a frequent adverse effect of VEGF-pathway inhibitors, including several tyrosine kinase inhibitors, a DASH-style pattern may be particularly relevant in selected oncology patients [25,170]. Nevertheless, its liver-specific evidence base remains less extensive than that of Mediterranean-style dietary patterns, and its application in the cardio-oncology setting is currently based on mechanistic and translational rationale rather than on direct interventional evidence.

Low-carbohydrate diets (LCDs) can reduce hepatic fat content through decreased de novo lipogenesis and improved insulin sensitivity, often within weeks and to a greater extent than conventional low-fat diets at comparable weight loss [172,173,174]. This effect is supported by short-term randomised controlled trials: benefits appear particularly pronounced in patients with obesity, insulin resistance or type 2 diabetes, who account for a large proportion of MASLD populations [174]. However, long-term adherence is frequently limited, and dietary quality may deteriorate when carbohydrate restriction is achieved through increased intake of saturated fats and processed animal products [175]. Current evidence therefore suggests that carbohydrate quality is at least as important as quantity: patterns emphasising minimally processed carbohydrates, legumes, vegetables and whole grains are more consistent with long-term cardiometabolic health than highly restrictive ketogenic approaches [160,173]. No international guideline currently endorses LCDs as first-line dietary therapy for MASLD, and no evidence is available in the cardio-oncology setting.

Intermittent fasting (IF) encompasses dietary strategies that alternate periods of food intake with periods of fasting or substantial energy restriction, whereas time-restricted feeding (TRF) limits daily food consumption to a defined eating window without necessarily prescribing an overall reduction in energy intake [176]. Both approaches have emerged as additional options for metabolic disease management. Randomised controlled trials suggest potential reductions in body weight, insulin resistance and hepatic fat, although it remains uncertain whether these effects are independent of reduced energy intake [177,178,179]. Within oncology, fasting-mimicking diets (FMDs) have also attracted interest because of their potential to improve chemotherapy tolerance and to reduce treatment-related toxicity through differential stress resistance mechanisms. FMDs are short-term, predominantly plant-based, low-energy regimens characterised by reduced protein and sugar intake and designed to reproduce selected metabolic effects of prolonged fasting, including reductions in insulin, insulin-like growth factor 1 and glucose signalling and increased ketogenesis, while permitting limited food consumption. However, clinical evidence remains preliminary and limited, and FMDs cannot currently be recommended for the prevention of treatment-related toxicity in cancer [180,181]. During active anticancer therapy, the maintenance of adequate energy and protein intake should remain the priority, particularly in patients at risk of malnutrition, cachexia or muscle loss [182,183]. Fasting-based strategies should therefore be considered only in carefully selected, nutritionally stable patients under specialist supervision.

Overall, no single dietary pattern is optimal for every patient with MASLD. Mediterranean-style dietary patterns provide the most comprehensive framework for simultaneously addressing liver health and cardiovascular risk, being the only pattern currently supported by both randomised interventional evidence and international guideline endorsement, whereas DASH, moderate carbohydrate restriction and time-restricted eating may be considered according to the dominant metabolic phenotype, treatment-related risks, nutritional status and patient preferences.

The strength of evidence supporting dietary interventions varies substantially according to the clinical outcome considered. The benefits of Mediterranean dietary patterns on cardiometabolic risk factors, cardiovascular prevention and MASLD are supported by prospective observational studies, randomised controlled trials, meta-analytical evidence and current international clinical guidelines. In contrast, evidence specifically supporting a role for dietary interventions in the prevention of CTR-CVT currently derives primarily from mechanistic and translational studies and from indirect extrapolation from cardiometabolic populations, whereas dedicated randomised controlled trials with CTR-CVT endpoints are lacking. Accordingly, dietary interventions should be regarded as evidence-based strategies for cardiometabolic optimisation and cardiovascular prevention, whereas their specific role in preventing CTR-CVT remains a translational hypothesis and an important area for future prospective investigation.

**Table 3:** Dietary interventions in MASLD and potential implications for cardio-oncology.

Intervention	Evidence in MASLD	Main Mechanisms	Potential Cardio-Oncology Implication
**Mediterranean diet**	Reduces liver fat and enzymes and cardiometabolic abnormalities [2,163,164,165,166,167,168,169].	Improved dietary quality, insulin sensitivity, mitochondrial function and endothelial NO bioavailability; reduces oxidative stress and inflammation burden	Guideline-supported foundational strategy for MASLD; direct CTR-CVT evidence remains limited
**DASH diet**	Improves liver enzymes, insulin resistance and inflammatory markers [170,171].	Blood pressure reduction, improved vascular function and reduced inflammation	Relevant in patients receiving VEGF inhibitors or TKIs; cardio-oncology use extrapolative
**Low-carbohydrate diets**	Rapid short-term reduction in hepatic fat, especially in obesity, insulin resistance and T2DM [172,173,174,175].	Reduced de novo lipogenesis and improved insulin sensitivity	Useful in selected patients; long-term quality and adherence require monitoring; no cardio-oncology evidence
**Intermittent fasting/time-restricted feeding**	Potential improvement in weight, insulin sensitivity and hepatic fat [176,177,178,179,184].	Autophagy, mitochondrial biogenesis, circadian alignment and metabolic flexibility	Investigational in oncology; avoid in malnutrition, cachexia or sarcopenic obesity

*Abbreviations*: DASH, Dietary Approaches to Stop Hypertension; MASLD, metabolic dysfunction-associated steatotic liver disease; T2DM, type 2 diabetes mellitus; TKI, tyrosine kinase inhibitor; VEGF, vascular endothelial growth factor.

### 7.2. Ultra-Processed Foods, the Microbiota, and the Gut–Liver Axis

The potential effects of nutritional interventions in MASLD may extend beyond energy balance and body weight. Diet influences gut microbial ecology, intestinal barrier function and the production of microbial metabolites, and may thereby contribute to hepatic and systemic metabolic homeostasis [11,185,186] (Table 4). Most evidence supporting gut–liver mechanisms is mechanistic or observational, and direct clinical evidence in cardio-oncology remains limited.

Ultra-processed foods (UPFs), as defined by the NOVA classification, are characterised by high contents of refined carbohydrates, added sugars, saturated fats and food additives together with low fibre and micronutrient density [187]. Their global consumption has increased markedly in parallel with the rising prevalence of obesity, type 2 diabetes and MASLD [188,189]. Large prospective cohort studies consistently associate higher UPF consumption with increased risks of MASLD, advanced fibrosis and cardiometabolic disease, independently of total caloric intake and body mass index [189,190,191]. Several mechanisms may explain these associations: added sugars and refined carbohydrates promote hepatic de novo lipogenesis, insulin resistance and postprandial hyperglycaemia [192]; food additives, including emulsifiers and artificial sweeteners, can alter gut microbial composition and disrupt the intestinal barrier in experimental models [193]. UPF consumption may therefore influence metabolic health not only through excessive energy intake, but also through effects on satiety regulation, microbial metabolites and immune signalling [189].

Among the components of UPFs, added fructose deserves particular attention. Following intestinal absorption, fructose is rapidly metabolised, predominantly in the liver at higher levels of intake, through ketohexokinase-C (KHK-C), thereby bypassing the phosphofructokinase-mediated regulatory step of glycolysis. Rapid fructose phosphorylation may induce ATP depletion and promote adenosine monophosphate degradation, with increased uric acid generation [192,194]. Fructose-derived substrates, together with activation of carbohydrate-responsive element-binding protein (ChREBP) and sterol regulatory element-binding protein-1c (SREBP-1c), stimulate hepatic de novo lipogenesis, triglyceride accumulation and hepatic insulin resistance, providing a biologically plausible link between excessive added-fructose intake and MASLD development and progression [192,194,195]. Higher consumption of sugar-sweetened beverages has been associated with hepatic steatosis, weight gain, obesity, type 2 diabetes and adverse cardiovascular outcomes [194,195,196]. These associations appear particularly relevant when fructose-containing sugars are consumed in liquid form or within an energy-surplus dietary pattern. The adverse metabolic effects attributed to added fructose should not be directly extrapolated to fructose naturally present in whole fruit, which is consumed within a matrix containing fibre, water and micronutrients. In oncology patients with MASLD, practical dietary counselling should therefore prioritise the reduction in sugar-sweetened beverages and fructose-rich ultra-processed foods, while preserving adequate energy and protein intake in individuals at risk of malnutrition, cachexia or sarcopenia. This approach should be integrated within the broader promotion of Mediterranean-style dietary patterns.

Gut dysbiosis has emerged as a key component of MASLD pathogenesis. Western dietary patterns reduce microbial diversity, decrease short-chain fatty acid (SCFA)-producing bacteria and increase intestinal permeability, facilitating portal translocation of lipopolysaccharide and other microbial-associated molecular patterns [185,197,198,199,200]. Subsequent activation of innate immune pathways stimulates Kupffer cells and hepatic stellate cells, promoting steatohepatitis, fibrogenesis and systemic inflammation [201]. These inflammatory signals may extend beyond the liver, contributing to endothelial dysfunction, insulin resistance and cardiovascular injury [19,185].

The gut microbiota also regulates host metabolism through bioactive metabolites. SCFAs improve gut barrier integrity, insulin sensitivity and immune homeostasis, whereas excessive production of trimethylamine (TMA) and its hepatic metabolite trimethylamine-N-oxide (TMAO) has been associated with endothelial dysfunction, atherosclerosis and adverse cardiovascular outcomes [202,203]. In addition, microbial regulation of bile acid metabolism modulates farnesoid X receptor (FXR) and Takeda G protein-coupled receptor 5 (TGR5)-dependent signalling pathways involved in glucose homeostasis, lipid metabolism and inflammation [204].

Beyond metabolic disease, the gut microbiota has emerged as a potential modulator of cancer treatment efficacy. Preclinical studies and emerging clinical observations suggest that microbial composition influences responses to immune checkpoint inhibitors, chemotherapy and radiotherapy through modulation of systemic immunity and inflammatory signalling [205,206]. Consequently, restoration of microbial diversity through dietary interventions may represent a common therapeutic strategy capable of simultaneously influencing hepatic disease progression, cardiovascular health and treatment tolerance.

Within this framework, Mediterranean dietary patterns appear particularly attractive because they combine high intake of dietary fibre, legumes, fruits, vegetables and extra-virgin olive oil, promoting SCFA production, preserving intestinal barrier integrity and reducing systemic inflammation [161,207]. Conversely, Western dietary patterns rich in ultra-processed foods favour gut dysbiosis, endotoxaemia and chronic inflammatory activation [197,198,199,200]. Nevertheless, the microbiota should be viewed as one potential mediator of dietary effects rather than as an established clinical endpoint. Dietary modulation of the gut–liver axis is biologically plausible, but its contribution to CTR-CVT prevention and treatment tolerance requires prospective validation.

**Table 4:** Gut microbiota-derived mechanisms linking nutrition, MASLD and cardio-oncology.

Gut-Derived Factor	Effect in MASLD	Cardiovascular Implication	Oncology Implication
**SCFAs**	Support intestinal barrier integrity and metabolic and immune homeostasis [202]	Potential improvement in insulin sensitivity and endothelial homeostasis	Potential improvement in immune regulation and modulation of immunotherapy response
**LPS/endotoxaemia**	Activation of hepatic innate immunity, Kupffer cells and stellate cells [197,198,199,200,201]	Endothelial dysfunction; systemic low-grade inflammation	Chronic inflammatory milieu potentially affecting tumour biology
**TMAO**	Marker of altered microbial–hepatic choline metabolism	Atherosclerotic and thrombotic pathways [203]	Clinical relevance remains uncertain
**Secondary bile acids**	FXR/TGR5 dysregulation; altered lipid and glucose homeostasis [204]	Metabolic and vascular dysfunction	Putative role in hepatocarcinogenesis through FXR-dependent signalling

*Abbreviations*: FXR, farnesoid X receptor; LPS, lipopolysaccharide; MASLD, metabolic dysfunction-associated steatotic liver disease; SCFAs, short-chain fatty acids; TGR5, Takeda G protein-coupled receptor 5; TMAO, trimethylamine-N-oxide.

### 7.3. Mediterranean Diet in Cancer Survivors: A Systems-Level Strategy Linking Liver, Cardiovascular and Metabolic Health

The growing population of cancer survivors has highlighted the need for interventions that simultaneously address metabolic dysfunction, cardiovascular risk and long-term sequelae of anticancer therapy. Survivors frequently exhibit a complex phenotype characterised by weight gain, insulin resistance, visceral adiposity, reduced physical activity, chronic inflammation and treatment-related metabolic derangements, all of which favour the development or progression of MASLD and cardiovascular disease [27,158]. These abnormalities often persist long after completion of oncological treatment and may strongly influence long-term outcomes.

Within this context, the Mediterranean diet represents a promising lifestyle strategies. Observational studies and meta-analyses report that greater adherence to the MD in cancer survivors is associated with lower cardiovascular mortality, reduced incidence of metabolic syndrome, lower systemic inflammation, improved endothelial function and favourable changes in body composition [28,29,168]. From a mechanistic perspective, components of the MD may influence multiple pathways implicated in both MASLD progression and CTR-CVT: extra-virgin olive oil provides monounsaturated fatty acids and polyphenols that reduce oxidative stress and improve endothelial NO bioavailability; fruits, vegetables and legumes supply antioxidants, fibre and phytochemicals that modulate inflammatory pathways and support gut microbial diversity; and marine-derived omega-3 fatty acids exert anti-inflammatory and anti-fibrotic actions while improving lipid metabolism and endothelial function [161,162,208]. Multidimensional lifestyle interventions that combine Mediterranean dietary counselling with structured exercise and cardiovascular risk factor optimisation have shown improvements in cardiorespiratory fitness, body composition, insulin sensitivity, blood pressure control and quality of life in cancer survivors, supporting the feasibility of integrating nutrition-based strategies into survivorship pathways [209,210]. Although direct randomised evidence specifically evaluating the prevention of cardiotoxicity in oncology patients is still lacking, these findings support the hypothesis that nutritional interventions may strengthen cardiovascular reserve before, during and after cancer treatment. Future prospective cardio-oncology studies should therefore incorporate hepatic endpoints, including liver fat quantification, non-invasive fibrosis biomarkers and liver stiffness measurement, to determine whether improvements in hepatic health contribute to the cardiovascular benefits of dietary interventions and to facilitate the development of precision nutrition strategies tailored to oncology patients with metabolic dysfunction.

### 7.4. Cardioprotective Nutraceuticals in the Oncology-MASLD Context

Beyond dietary patterns, increasing attention has been directed toward specific nutraceuticals capable of modulating the metabolic, inflammatory and oxidative pathways shared by MASLD, cardiovascular disease and CTR-CVT. Nutritional supplementation cannot substitute for comprehensive lifestyle interventions, but selected compounds may provide adjunctive benefits in carefully selected patients, particularly in the presence of documented deficiencies or specific metabolic abnormalities (Table 5). However, the strength of evidence supporting these interventions varies considerably, ranging from mechanistic and preclinical studies to observational clinical evidence, randomised controlled trials and current guideline recommendations.

Among currently available nutraceuticals, omega-3 polyunsaturated fatty acids (PUFAs) possess the strongest evidence base. Experimental studies have consistently demonstrated that eicosapentaenoic acid (EPA) and docosahexaenoic acid (DHA) reduce hepatic triglyceride accumulation, improve lipid metabolism and exert anti-inflammatory effects through modulation of nuclear receptors, eicosanoid synthesis and inflammatory signalling [161,208]. Clinical evidence from randomised controlled trials and meta-analyses in MASLD have shown reductions in liver fat content and serum triglycerides after omega-3 supplementation, with less consistent effects on fibrosis [211,212]; from a cardiovascular perspective, clinical studies suggest that PUFAs may improve endothelial function and reduce vascular inflammation, with potential dose-dependent benefits on cardiovascular outcomes in selected populations [213]. Their biological actions overlap with several pathways implicated in anthracycline-induced oxidative stress and endothelial dysfunction, supporting further investigation within the cardio-oncology setting. Current MASLD guidelines do not recommend routine omega-3 supplementation as disease-specific therapy, although it may be considered for the management of hypertriglyceridaemia [2].

Vitamin D deficiency is highly prevalent in patients with obesity, MASLD and cancer. Beyond bone metabolism, experimental evidence suggests that vitamin D may regulate immune responses, inflammatory signalling and endothelial function [214,215]. Observational studies have linked low circulating concentrations to greater MASLD severity and increased cardiovascular risk [216]. However, randomised intervention studies have produced mixed results and current evidence does not support routine vitamin D supplementation for MASLD treatment in the absence of deficiency, although correction of documented deficiency remains clinically appropriate [2,214,215].

Magnesium, selenium and zinc participate in glucose metabolism, mitochondrial ATP production, vascular homeostasis and antioxidant defence through glutathione peroxidase and superoxide dismutase activity [217,218,219]. Observational studies have associated lower magnesium intake has been associated with insulin resistance, type 2 diabetes and increased cardiovascular risk, and selenium and zinc deficiencies with oxidative stress and immune dysregulation [218,219]. Routine supplementation specifically for MASLD regression or cardiotoxicity prevention is not currently supported by robust evidence; supplementation should be individualised and primarily guided by documented deficiencies or increased requirements.

L-carnitine plays a central role in mitochondrial fatty acid transport and β-oxidation. Experimental studies suggest that carnitine supplementation may improve mitochondrial efficiency, reduce oxidative stress and attenuate hepatic lipid accumulation [220], and randomised trials in non-alcoholic steatohepatitis have reported improvements in liver enzymes and metabolic parameters, although findings remain heterogeneous [221]. Interest in L-carnitine has also emerged in cardio-oncology because of its potential to counteract the mitochondrial dysfunction that underlies anthracycline cardiotoxicity, but current evidence is still insufficient to recommend routine supplementation.

Glycine has recently attracted interest because of its anti-inflammatory, cytoprotective and mitochondrial-supportive properties, with experimental models suggesting attenuation of hepatic inflammation, oxidative stress and mitochondrial dysfunction [222]. Clinical evidence remains preliminary, and glycine should currently be considered an investigational adjunct rather than an established therapeutic strategy.

Overall, nutraceutical interventions should be viewed as complementary components of a broader nutritional strategy centred on Mediterranean dietary patterns, physical activity and metabolic risk factor optimisation. While several compounds show promising biological effects in experimental models, the level of evidence differs substantially across interventions, and for most nutraceuticals remains limited to preclinical studies, observational data or small randomised clinical trials. Consequently, current international guidelines support supplementation mainly in the presence of documented deficiencies or specific clinical indications rather than routine use for MASLD or cardio-oncology prevention.

**Table 5:** Nutraceuticals with potential relevance in the MASLD–Cardio-Oncology Triangle.

Nutraceutical	Main Biological Target	Evidence in MASLD	Potential Cardio-Oncology Implication
**Omega-3 PUFAs (EPA/DHA)**	Inflammation, lipid metabolism, endothelial function	Reduces liver fat and triglycerides, less consistent effects on fibrosis [161,208,211,212,213]	Potential adjunctive strategy; direct evidence for CTR-CVT prevention remains limited
**Vitamin D**	Immune regulation, endothelial function, inflammatory signalling	Benefit limited to patients with documented deficiency [214,215,216]	Correct documented deficiency; no routine supplementation
**Magnesium**	Insulin sensitivity, mitochondrial ATP production, vascular homeostasis	Observational evidence linking low intake to insulin resistance and CV risk [217,218,219]	Deficiency-guided supplementation
**Selenium**	Antioxidant defence (glutathione peroxidase)	Limited clinical evidence; deficiency linked to oxidative stress [219]	Individualised use based on documented deficiency
**Zinc**	Antioxidant and immune function (superoxide dismutase)	Limited clinical evidence; deficiency linked to immune dysregulation [217,218,219]	Individualised use based on documented deficiency
**L-carnitine**	Mitochondrial fatty acid β-oxidation	Heterogeneous RCT evidence in NASH; improvements in liver enzymes and metabolic parameters [220,221]	Investigational; potential counteraction of anthracycline-related mitochondrial dysfunction
**Glycine**	Anti-inflammatory and mitochondrial-supportive properties	Preliminary experimental evidence [222]	Investigational adjunct; insufficient clinical evidence

*Abbreviations*: ATP, adenosine triphosphate; CV, cardiovascular; DHA, docosahexaenoic acid; EPA, eicosapentaenoic acid; MASLD, metabolic dysfunction-associated steatotic liver disease; NASH, non-alcoholic steatohepatitis; PUFAs, polyunsaturated fatty acids; RCT, randomised controlled trial.

### 7.5. Management of Sarcopenic Obesity: Protein Adequacy, Leucine, Resistance Exercise, Nutritional Timing

Sarcopenic obesity has emerged as a clinically relevant phenotype at the intersection of MASLD, oncology and cardiovascular disease. Characterised by the coexistence of excess adiposity and reduced skeletal muscle mass or function, it has been associated with metabolic dysfunction, chemotherapy intolerance, physical disability and adverse clinical outcomes [153,154,223]. In MASLD, skeletal muscle is a major regulator of glucose disposal and insulin sensitivity, and progressive muscle loss may aggravate insulin resistance, hepatic steatosis and systemic inflammation, generating a self-perpetuating cycle that may accelerate disease progression [153,157]. In oncology populations, sarcopenia is associated with increased treatment-related toxicity, more frequent dose reductions, poorer quality of life and reduced survival [155,156].

Preservation of skeletal muscle mass requires adequate protein intake, particularly during periods of metabolic stress such as cancer treatment. ESPEN guidelines and subsequent evidence suggest that protein requirements in patients with cancer and metabolic dysfunction frequently exceed those for healthy adults, with daily intakes of approximately 1.2–1.5 g/kg body weight often proposed to support muscle protein synthesis and functional recovery [182]. Protein quality is equally important: high-biological-value proteins rich in essential amino acids stimulate muscle protein synthesis more effectively than lower-quality sources [224,225]. Leucine, a branched-chain amino acid that activates the mTOR pathway, plays a key role in this anabolic response and may be particularly useful in older adults or patients with anabolic resistance, especially when combined with adequate total protein intake and exercise [225,226].

Resistance exercise remains the most effective non-pharmacological intervention for preventing and reversing sarcopenia. In both MASLD and cancer populations, resistance training improves muscle mass, strength, insulin sensitivity and physical function, while reducing visceral adiposity and systemic inflammation [160,227]. Its benefits extend beyond skeletal muscle to include improvements in hepatic steatosis, mitochondrial function, endothelial health and cardiorespiratory fitness, supporting structured resistance training as a core component of any integrated strategy targeting the MASLD–Cardio-Oncology Triangle [160]. Emerging evidence further suggests that the timing of nutrient intake influences anabolic responses: protein consumption distributed across meals and provided in proximity to exercise sessions appears to maximise muscle protein synthesis and recovery [224,228], while synchronisation of feeding patterns with circadian rhythms may improve metabolic flexibility, insulin sensitivity and mitochondrial function. The management of sarcopenic obesity is a key component of personalised nutrition in oncology patients with MASLD, given its links with metabolic dysfunction, treatment tolerance and cardiovascular vulnerability. Prospective studies are needed to determine whether preserving skeletal muscle mass and function can reduce CTR-CVT and improve long-term outcomes.

**Figure 2:**
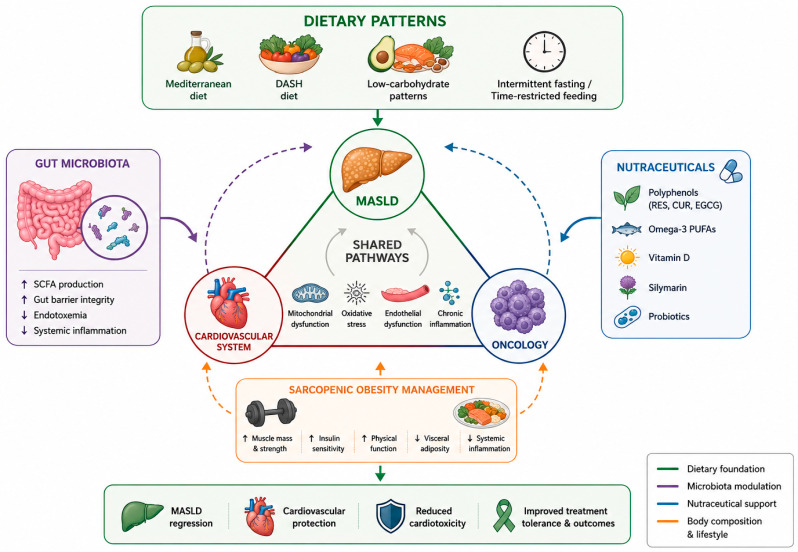
Diet, microbiota, nutraceuticals and sarcopenic obesity as modifiers of the MASLD–Cardio-Oncology Triangle. *Abbreviations*: DASH, Dietary Approaches to Stop Hypertension; EGCG, epigallocatechin gallate; CUR, curcumin; MASLD, metabolic dysfunction-associated steatotic liver disease; PUFA, polyunsaturated fatty acid; RES, resveratrol; SCFA, short-chain fatty acid. *Symbols*: ↑, increased; ↓, decreased.

Collectively, Mediterranean-style dietary patterns, gut microbiota modulation, selected nutraceuticals and management of sarcopenic obesity may act on multiple shared pathways of the MASLD–Cardio-Oncology Triangle, with the potential to influence hepatic, cardiovascular and oncological outcomes (Figure 2). However, evidence for integrated benefits within oncology cohorts with documented MASLD remains limited.

## 8. Pharmacotherapies Targeting the MASLD–Cardio-Oncology Triangle

Pharmacological strategies relevant to the MASLD–Cardio-Oncology Triangle span two complementary fronts: liver-directed agents of new generation, including the thyroid hormone receptor-β (THR-β) agonist resmetirom and emerging FGF21 analogues, and cardiometabolic drugs, including statins, metformin, glucagon-like peptide-1 receptor agonists (GLP-1RAs) and sodium-glucose cotransporter-2 inhibitors (SGLT2i), long established for cardiovascular and metabolic indications and now investigated for chemopreventive potential. Although most available evidence remains observational, several of these therapies show converging signals across hepatic, cardiovascular and oncological domains (Table 6). The evidence is heterogeneous: randomised trials support selected hepatic or cardiometabolic endpoints, whereas most cancer-related signals remain observational or hypothesis-generating.

### 8.1. Resmetirom and the THR-β Pathway

The well-established associations between hypothyroidism, metabolic syndrome and insulin resistance prompted the development of liver-selective thyroid hormone receptor-β (THR-β) agonists, culminating in the accelerated approval of resmetirom as the first pharmacological therapy specifically indicated for MASH [229,230]. In the phase 3 MAESTRO-NASH trial, resmetirom achieved both primary endpoints at 52 weeks: MASH resolution without fibrosis worsening and ≥1-stage fibrosis improvement without MASH worsening [231]. Mechanistically, THR-β activation promotes hepatic fatty acid β-oxidation, suppresses lipogenic genes, and enhances LDL receptor expression, thereby reducing LDL cholesterol, an effect with potential cardiovascular relevance in MASH [232,233,234]. Preclinical data also suggest anti-inflammatory effects through the downregulation of TNF-α and IL-1β, and attenuation of hepatic stellate cell activation [232]. However, the available evidence has important limitations. Current phase 3 data primarily demonstrate histological and biochemical efficacy at 52 weeks, whereas long-term clinical outcomes remain incompletely characterised. The trials conducted to date were not designed or adequately powered to determine whether resmetirom reduces major adverse cardiovascular events, heart failure, cardiovascular mortality, cancer incidence, cancer recurrence or cancer therapy-related cardiovascular toxicity. Although the observed reduction in LDL cholesterol may have potential cardiovascular relevance, improvement in a surrogate lipid marker should not be interpreted as evidence of cardiovascular event reduction. Moreover, dedicated oncological and cardio-oncology studies are currently lacking. Therefore, any potential cardiovascular or oncological benefit of resmetirom remains hypothesis-generating and requires confirmation in long-term prospective studies incorporating clinically relevant hepatic, cardiovascular and oncological outcomes.

### 8.2. FGF21 Analogues

Fibroblast growth factor 21 (FGF21) analogues represent a second class of liver-directed agents in advanced clinical development for MASH [85]. FGF21 reduces hepatic de novo lipogenesis, enhances fatty acid β-oxidation and improves insulin sensitivity and lipid profile, with mechanisms potentially relevant across the MASLD–Cardio-Oncology Triangle. Two analogues have produced the most robust evidence: efruxifermin, which improved fibrosis in patients with F2-F3 MASH [235] and is being evaluated in compensated cirrhosis [236]; and pegozafermin, which met both fibrosis improvement and MASH resolution endpoints in F2-F3 MASH [237]. Both consistently improved HbA1c, triglycerides and atherogenic lipid markers, suggesting potential translational relevance to cardiovascular and oncological risk, although dedicated cardio-oncology data remain unavailable. Phase 3 programmes are ongoing.

### 8.3. GLP-1 Receptor Agonists

GLP-1RAs act predominantly through indirect, weight- and glucose-mediated pathways, since canonical GLP-1 receptor expression in hepatocytes is very low [238]. Inhibition of enterocyte chylomicron secretion, reduction in VLDL, improvement of glucose homeostasis and weight loss collectively contribute to the hepatoprotective and cardiometabolic effects of this class [239,240,241]. In a phase 2 trial of 320 patients with biopsy-confirmed MASH, once-daily subcutaneous semaglutide significantly increased the proportion achieving MASH resolution without worsening of fibrosis, with parallel improvements in body weight and glycated haemoglobin [242]. A separate phase 2 trial in compensated MASH cirrhosis confirmed favourable cardiometabolic effects without new safety signals [243]. The phase 3 ESSENCE trial subsequently demonstrated that once-weekly semaglutide 2.4 mg significantly increased steatohepatitis resolution at interim analysis [244]. Beyond histology, large observational studies have associated GLP-1RA use, particularly when combined with metformin [245], with lower incidence of obesity-related malignancies, including HCC, colorectal, pancreatic and endometrial cancers [246,247,248], although continued pharmacovigilance for thyroid and possibly kidney cancer is warranted [249].

The cardiovascular benefits of GLP-1RAs are supported by large randomised cardiovascular outcome trials demonstrating reductions in MACE in selected patients with type 2 diabetes, obesity, or established cardiovascular disease. These effects should be distinguished from their potential role in cardio-oncology, for which the evidence remains preliminary. Observational studies reporting lower rates of obesity-related malignancies provide hypothesis-generating oncological signals but do not establish causality. Moreover, dedicated prospective trials have not yet determined whether GLP-1RAs prevent CTR-CVT, improve tolerance or completion of anticancer therapy, reduce treatment interruptions, or influence cancer recurrence and survival. Therefore, their current use in patients with cancer should be guided by established metabolic and cardiovascular indications, while any specific cardio-oncological benefit remains an emerging hypothesis requiring prospective validation.

### 8.4. Statins

Among currently available cardiometabolic agents, statins show the most consistent chemopreventive signal. Several meta-analyses have reported lower HCC incidence among statin users, with stronger associations for lipophilic statins and higher cumulative doses [250,251]; a 2026 analysis, specifically in MASLD, confirmed reductions in HCC incidence, all-cause mortality and liver-related mortality among statin-treated patients [252]. Beyond lipid lowering, statins modulate cholesterol synthesis, inflammation, endothelial function and cell proliferation through inhibition of the mevalonate pathway, providing a plausible biological rationale for their antitumour activity [253]. These effects complement the well-established cardiovascular benefits of statins [254,255], making them particularly attractive in patients with MASLD and elevated cardiovascular risk [256,257]. Nevertheless, the absence of randomised trials specifically designed to evaluate cancer prevention precludes definitive causal conclusions.

### 8.5. Metformin

Metformin has long been considered a potential anticancer agent because of its effects on insulin resistance, AMPK activation and cellular metabolism, but its role remains uncertain. Although observational data suggest an approximately 18% reduction in MASLD-related HCC, randomised trials and propensity-score-matched meta-analyses have shown no reduction in overall cancer incidence [257,258]. Current evidence therefore supports metformin primarily as a treatment for type 2 diabetes rather than as a chemopreventive strategy per se.

### 8.6. SGLT2 Inhibitors

SGLT2 inhibitors have established cardiovascular and renal benefits supported by large randomised cardiovascular outcome trials, particularly in patients with heart failure, chronic kidney disease and type 2 diabetes. These proven effects are clinically relevant for the management of cardiovascular comorbidities in patients with cancer but should not be interpreted as direct evidence of protection against CTR-CVT. Emerging observational studies in populations with MASLD and type 2 diabetes, including two large multinational cohorts and a recent meta-analysis, have reported a lower risk of HCC among SGLT2 inhibitor users [259,260,261].

By contrast, randomised cardiovascular outcome trials have shown an overall neutral effect on cancer incidence, supporting the oncological safety of this drug class rather than establishing a definitive chemopreventive benefit. Preliminary mechanistic and translational evidence suggests that SGLT2 inhibition may improve myocardial energetics, reduce inflammation and oxidative stress, and potentially enhance cardiac resilience during anticancer therapy. However, dedicated randomised clinical trials evaluating CTR-CVT, tolerance to anticancer treatment, treatment completion, cancer recurrence or survival are currently lacking. Accordingly, SGLT2 inhibitors should presently be prescribed according to their established cardiovascular, renal and glycaemic indications, whereas their specific role in cardio-oncology should still be regarded as investigational.

Taken together, the pharmacological strategies discussed in this section differ substantially in both therapeutic target and level of supporting evidence (Table 6). Liver-directed agents, including resmetirom and FGF21 analogues, have demonstrated histological and metabolic benefits in MASH, whereas GLP-1 receptor agonists and SGLT2 inhibitors have robust cardiovascular and metabolic efficacy in appropriately selected non-oncological populations. Statins currently provide the strongest observational evidence for a possible reduction in HCC risk. By contrast, evidence regarding CTR-CVT prevention, anticancer treatment tolerance and oncological outcomes remains predominantly observational, retrospective, preclinical or mechanistic. Until dedicated randomised trials with clinically relevant hepato-cardio-oncological endpoints become available, these agents should be prescribed according to established clinical indications rather than specifically for cancer prevention or protection against anticancer therapy-related cardiovascular toxicity.

**Table 6:** Pharmacotherapies across the MASLD–Cardio-Oncology Triangle.

Drug Class	Evidence in MASLD	Main Mechanisms	Potential Cardio-Oncology Implication
**Resmetirom (THR-β agonist)**	MASH resolution and fibrosis improvement [229,231]	Hepatic β-oxidation, suppression of lipogenic genes, LDL receptor upregulation [232,233,234]	LDL cholesterol reduction with potential CV
**FGF21 analogues (efruxifermin, pegozafermin)**	Fibrosis improvement and MASH resolution [85,235,236,237]	Reduced hepatic de novo lipogenesis; improved insulin sensitivity, adiponectin and atherogenic lipid markers	Favourable cardiometabolic profile; oncological outcomes not yet evaluated
**Statins**	Reduced HCC incidence and liver-related mortality in MASLD [250,251,252,255]	Mevalonate pathway inhibition; anti-inflammatory, antiproliferative and endothelial effects [253]	Established CV benefit; consistent chemopreventive signal in observational data, RCT confirmation pending
**Metformin**	Modest hepatic effect; reduced MASLD-related HCC in observational data [257]	AMPK activation, reduced insulin resistance	No reduction in overall cancer incidence in RCTs and PSM meta-analyses [258]
**GLP-1 receptor agonists**	MASH resolution in phase 2/3 trials [238,239,240,241,242,243,244]	Weight loss, improved glucose homeostasis, reduced VLDL and chylomicron secretion	Reduced MACE; lower incidence of obesity-related cancers in observational studies [245,246,247,248]; potential benefits for cardio-oncology and cancer therapy tolerance remain investigational
**SGLT2 inhibitors**	Lower HCC risk in MASLD–T2DM cohorts [259,260,261]	Glycosuria, weight loss, improved cardiac and renal haemodynamics	Established CV and renal protection; effects on CTR-CVT and cancer therapy tolerance have yet to be evaluated

*Abbreviations*: CV, cardiovascular; FGF21, fibroblast growth factor 21; GLP-1, glucagon-like peptide-1; HCC, hepatocellular carcinoma; LDL, low-density lipoprotein; MACE, major adverse cardiovascular events; MASH, metabolic dysfunction-associated steatohepatitis; MASLD, metabolic dysfunction-associated steatotic liver disease; PSM, propensity-score-matched; RCT, randomised controlled trial; SGLT2, sodium-glucose cotransporter-2; THR-β, thyroid hormone receptor-β; T2DM, type 2 diabetes mellitus; VLDL, very-low-density lipoprotein.

## 9. Clinical Implications and Integrated Framework

The convergence of metabolic, cardiovascular and oncological pathways outlined in the previous sections has potential implications for clinical practice. MASLD may be considered not only as a hepatic condition but also as a systemic metabolic disorder associated with cardiovascular vulnerability and cancer risk, while its influence on tolerance to anticancer therapies remains uncertain. Translating this concept into routine care requires prospective validation of a structured operational framework, capable of identifying high-risk patients before treatment; of monitoring the relevant pathophysiological axes during therapy; and of integrating nutritional, cardiometabolic and oncological expertise into shared decision-making [2,9,19].

### 9.1. Proposed Clinical Algorithm

**Figure 3:**
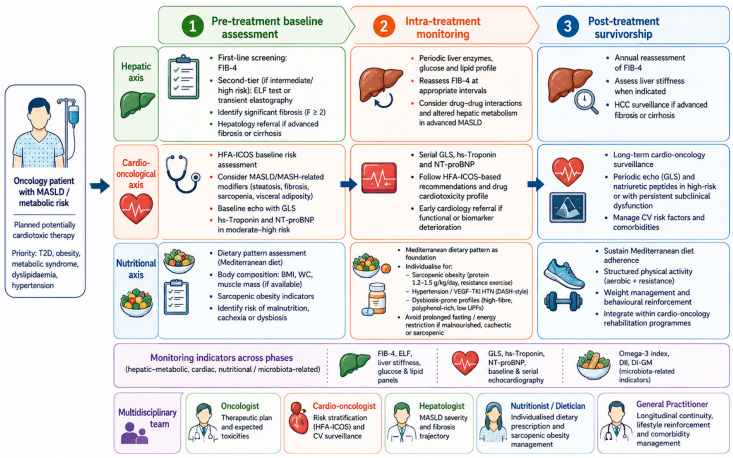
Integrated MASLD–Cardio-Onco–Nutritional clinical pathway across the cancer care continuum. *Abbreviations*: BMI, body mass index; CV, cardiovascular; DASH, Dietary Approaches to Stop Hypertension; DII, Dietary Inflammatory Index; DI-GM, gut microbiota dysbiosis index; ELF, Enhanced Liver Fibrosis; FIB-4, Fibrosis-4 index; GLS, global longitudinal strain; HCC, hepatocellular carcinoma; HFA-ICOS, Heart Failure Association-International Cardio-Oncology Society; hs-Troponin, high-sensitivity troponin; HTN, hypertension; MASLD, metabolic dysfunction-associated steatotic liver disease; MASH, metabolic dysfunction-associated steatohepatitis; NT-proBNP, N-terminal pro-B-type natriuretic peptide; T2D, type 2 diabetes; TKI, tyrosine kinase inhibitor; UPFs, ultra-processed foods; VEGF, vascular endothelial growth factor; WC, waist circumference.

Within the proposed MASLD–Cardio-Oncology Triangle, three sequential steps may be considered (Figure 3).

First, baseline MASLD assessment may be considered in patients scheduled to receive potentially cardiotoxic regimens, particularly those with type 2 diabetes, obesity, metabolic syndrome or dyslipidaemia. A pragmatic non-invasive approach, consistent with current European guidelines, combines FIB-4 as a first-line tool with a second-tier evaluation using the Enhanced Liver Fibrosis (ELF) test or transient elastography (vibration-controlled or two-dimensional shear-wave elastography) to identify clinically significant fibrosis (F ≥ 2) [2,3]. Patients with intermediate or high-risk scores should be referred to hepatology for further characterisation before treatment initiation. This proposed extension of hepatic phenotyping to cardio-oncology has not yet been prospectively validated.

Second, cardio-oncological stratification should be performed according to the HFA-ICOS risk assessment tools, while MASLD-/MASH-related metabolic parameters (steatosis, fibrosis stage, sarcopenia and visceral adiposity) may be recorded as exploratory modifiers alongside existing risk categories [24,25,26,131]. Although MASLD is not yet a formal item of the HFA-ICOS score, its frequent coexistence with established risk factors and its biological overlap with mechanisms driving CTR-CVT support its consideration as an emerging amplifier of cardiovascular vulnerability in cardio-oncology decision-making [19,78].

Third, individualised nutritional assessment and prescription may be embedded in baseline evaluation and follow-up, when clinically indicated. The Mediterranean diet represents a guideline-supported dietary pattern for MASLD and cardiovascular risk reduction, whereas oncology-specific evidence remains less direct [29,160,163,164,165]. Specific adaptations may be considered for patients with sarcopenic obesity (protein optimisation, leucine-enriched foods, resistance exercise) [153,154,225,228], for hypertension or VEGF/TKI-induced hypertension (DASH-style modifications) [170,171], or for markers of dysbiosis (high-fibre, polyphenol-rich, low-UPF strategies) [186,193,207]. Energy-restrictive regimens and prolonged fasting protocols should be avoided in patients with established malnutrition, cachexia or sarcopenia [181,182].

### 9.2. Monitoring Indicators

Beyond conventional laboratory parameters, a clinically actionable monitoring set may include: (i) hepatic-metabolic indicators (FIB-4, ELF, liver stiffness, where available); (ii) cardiac surveillance (GLS on echocardiography, high-sensitivity troponins, NT-proBNP) according to HFA-ICOS-based recommendations [25,26]; and (iii) nutritional and microbiota-related biomarkers (omega-3 index, Dietary Inflammatory Index [DII] and the more recent Dietary Index for Gut Microbiota [DI-GM]) [208,262,263]. Although several of these biomarkers are not yet routine, they provide a coherent translational platform for the integrated monitoring of the hepato-cardio-oncological continuum.

### 9.3. The Role of the Multidisciplinary Team

The complexity of the MASLD–Cardio-Oncology Triangle cannot be effectively addressed within a single speciality. A multidisciplinary team, including an oncologist, cardiologist, hepatologist, clinical nutritionist or dietician, and general practitioner, is required to coordinate pre-treatment assessment, intra-treatment monitoring and post-treatment surveillance. Each professional contributes specific competencies: oncology defines the therapeutic plan and its expected toxicities; cardio-oncology applies risk scores and structures cardiovascular surveillance [25]; hepatology assesses MASLD severity and fibrosis trajectory [2]; nutrition translates dietary evidence into individualised prescriptions and monitors sarcopenic obesity [182,191,225]; and the general practitioner ensures longitudinal continuity, lifestyle reinforcement and management of comorbidities. Future survivorship pathways should integrate all four domains within shared cardio-onco–hepato–nutritional clinics whenever feasible.

### 9.4. Key Take-Home Messages

- Screen every patient scheduled for cardiotoxic therapy with FIB-4 at baseline; refer those with intermediate or high scores for elastography or ELF.
- Stratify cardiovascular risk using HFA-ICOS, considering MASLD/MASH as an additional modifier in patients with metabolic dysfunction.
- Prescribe a Mediterranean dietary pattern, individualised for sarcopenic obesity, hypertension or dysbiosis-prone profiles.
- Avoid prolonged fasting or energy-restrictive regimens in patients at risk of malnutrition or sarcopenia.
- Monitor GLS, high-sensitivity troponins and NT-proBNP during and after treatment; reassess FIB-4 annually.
- Refer to a multidisciplinary cardio-onco-hepato-nutritional pathway whenever the metabolic, cardiac and oncological domains intersect.

Future prospective studies will be required to confirm whether structured integration of MASLD assessment within cardio-oncology pathways translates into improved cardiovascular and oncological outcomes, but the biological rationale and the convergence of current evidence support its consideration as a pragmatic step towards precision survivorship care.

## 10. Limitations and Evidence Gaps

Despite the increasing recognition of MASLD as a systemic metabolic disorder with cardiovascular and oncological implications, the evidence supporting its integration into cardio-oncology pathways remains fragmented and predominantly indirect. Several limitations should therefore be acknowledged when interpreting the framework proposed in this review (Table 7).

First and most fundamentally, no prospective clinical study has directly evaluated MASLD as an independent predictor or effect modifier of cancer therapy-related cardiovascular toxicity. The available evidence derives from two largely parallel bodies of literature: observational cohorts of patients with diabetes, obesity or NAFLD/MASLD followed primarily for cardiovascular or hepatic outcomes, in which oncological endpoints are generally secondary or assessed post hoc [16,17,18,108]; and cardio-oncology studies that have rarely incorporated systematic hepatic phenotyping [24,26]. Accordingly, the MASLD–Cardio-Oncology Triangle is currently supported by biological plausibility and indirect observational evidence rather than by direct clinical validation. Its proposed clinical implications should therefore be regarded as hypothesis-generating until confirmed in dedicated prospective studies.

Second, substantial heterogeneity exists in disease definitions and diagnostic methods across studies. Many trials and registries continue to use the former NAFLD nomenclature or rely on imaging-based definitions, including ultrasonography, hepatic steatosis indices or controlled attenuation parameter, without histological or elastographic characterisation of fibrosis. Moreover, the 2023 multisociety MASLD nomenclature and the 2024 EASL-EASD-EASO criteria have only recently been adopted [1,2]. This nosological and methodological inconsistency complicates direct comparison and meta-analytic pooling of existing data and limits the external validity of any unified risk model.

Third, the strong confounding effect of obesity, insulin resistance, type 2 diabetes, sarcopenia and visceral adiposity is difficult to disentangle. MASLD shares its principal determinants with these conditions, and most cardiovascular and oncological associations are likely to reflect overlapping rather than fully independent mechanisms [8,17,30,157]. The relative contribution of liver-specific pathways remains incompletely defined, particularly in oncological cohorts. Reverse causality also cannot be excluded, as cancer-related inflammation, treatment-related metabolic changes, weight fluctuations and muscle loss may contribute to or worsen hepatic steatosis and metabolic dysfunction.

Fourth, composite hepato-cardio-oncological endpoints are currently lacking. Few studies have integrated MACE, liver-related events (decompensation, hepatocellular carcinoma) and cancer-related outcomes (recurrence, treatment interruptions, mortality) within a unified statistical framework. The absence of standardised composite endpoints hinders the design of pragmatic trials and the development of integrated risk scores tailored to the MASLD–Cardio-Oncology Triangle.

Finally, the present work is a narrative review and, as such, is intrinsically susceptible to selective citation, the absence of pre-registered systematic review protocols, and the lack of formal risk-of-bias assessment and quantitative evidence synthesis. Preregistered systematic reviews, individual patient-data meta-analyses and umbrella reviews will be necessary to consolidate the proposed conceptual framework and determine whether it can be translated into clinically validated tools.

**Table 7:** Key limitations of current evidence and corresponding future research priorities in the MASLD–Cardio-Oncology field.

Key Limitation of Current Evidence	Interpretative or Clinical Consequence	Corresponding Future Research Priority
**Absence of direct clinical evidence linking MASLD to CTR-CVT**	The proposed association remains biologically plausible but clinically unproven; the independent and incremental prognostic value of MASLD beyond established cardio-oncology risk factors cannot currently be determined.	Prospective cardio-oncology cohorts explicitly incorporating baseline non-invasive hepatic phenotyping, longitudinal cardiovascular surveillance and adjudicated CTR-CVT outcomes.
**Heterogeneity of MASLD definitions and diagnostic methods**	Inconsistent nomenclature and variable diagnostic approaches reduce comparability across studies, complicate evidence synthesis and limit external validity.	Uniform application of the 2023 multisociety MASLD nomenclature and the 2024 EASL-EASD-EASO diagnostic and risk-stratification criteria in future studies.
**Residual confounding and reverse causality**	Shared metabolic and body-composition determinants make it difficult to establish whether MASLD independently contributes to CTR-CVT or primarily reflects cumulative cardiometabolic vulnerability; cancer and its treatments may also induce or worsen MASLD-related phenotypes.	Prospective cohorts incorporating baseline and serial hepatic and body-composition phenotyping, treatment-specific CTR-CVT adjudication, and analyses distinguishing confounding, mediation, interaction and temporal directionality.
**Absence of standardised composite hepato-cardio-oncological endpoints**	CTR-CVT, MACE, liver-related events, treatment tolerance and oncological outcomes are generally evaluated separately, limiting integrated assessment.	Development of consensus composite endpoints integrating CTR-CVT, liver-related events, treatment interruptions or dose reductions, and oncological outcomes.
**Lack of interventional evidence in MASLD-positive oncology cohorts**	Nutritional and pharmacological interventions have not been specifically evaluated in this population, and their effects on CTR-CVT and treatment tolerance remain uncertain.	Peri-treatment interventional trials evaluating Mediterranean-style dietary patterns, protein-optimised and exercise-based strategies for sarcopenic obesity, and selected metabolically targeted therapies.
**Intrinsic limitations of a narrative review**	The evidence synthesis is susceptible to selective citation and is not supported by a pre-registered systematic search protocol or formal risk-of-bias assessment.	Preregistered systematic reviews, umbrella reviews and individual participant-data meta-analyses addressing the MASLD–Cardio-Oncology field.

*Abbreviations*: CTR-CVT, cancer therapy-related cardiovascular toxicity; EASL-EASD-EASO, European Association for the Study of the Liver–European Association for the Study of Diabetes–European Association for the Study of Obesity; MACE, major adverse cardiovascular events; MASLD, metabolic dysfunction-associated steatotic liver disease.

## 11. Future Directions

Several research priorities emerge from the limitations outlined above. Multicentre intervention trials of peri-chemotherapy nutritional strategies in MASLD-positive oncology cohorts are needed to test whether Mediterranean-style dietary patterns, sarcopenia-targeted protein optimisation and individualised counselling can modulate cardiotoxicity, treatment tolerance and quality of life in patients receiving anthracyclines, HER2-targeted therapies, VEGF/TKI agents or immune checkpoint inhibitors [165,179,182,183].

In parallel, composite endpoints integrating MACE, liver-related events and cancer recurrence or mortality should become standard in cardio-oncology research, allowing simultaneous quantification of the cardiovascular, hepatic and oncological dimensions of treatment-related morbidity. Such endpoints would directly support the development and prospective validation of integrated risk tools, such as a MASLD–Cardio-Onco Risk Score, combining HFA-ICOS items, non-invasive fibrosis markers (FIB-4, ELF, liver stiffness), sarcopenia indices and nutritional parameters [24,26,131].

Chemopreventive trials with metabolic-targeted therapies, including resmetirom in MASH, GLP-1 receptor agonists, dual or triple incretin agonists, FGF21 analogues and SGLT2 inhibitors, should explicitly enrol patients with MASLD at high oncological risk, evaluating both hepatic and extrahepatic cancer endpoints [229,231,238,244,246,247,252,257,259]. These molecules act on shared metabolic pathways linking MASLD, cardiovascular disease and tumour biology, and represent natural candidates to test the chemopreventive arm of the proposed triangle.

Finally, precision nutrition strategies based on multi-omics profiling (metabolomics, lipidomics, proteomics, microbiomics) and on dynamic dietary algorithms supported by artificial intelligence may allow real-time adaptation of nutritional plans across the cancer care continuum. Coupled with digital phenotyping of physical activity, body composition and adherence, such approaches could move cardio-onco–nutritional care towards a genuinely personalised paradigm.

## 12. Conclusions

MASLD has rapidly emerged as one of the most prevalent metabolic disorders worldwide, and its biological footprint extends well beyond the liver. As discussed throughout the present review, MASLD is associated with biological pathways that also feature in cardiovascular disease and several oncological conditions: insulin resistance, chronic low-grade inflammation, oxidative and nitrosative stress, mitochondrial dysfunction, lipotoxicity, dysbiosis and immune dysregulation collectively, which shape a systemic milieu that may favour cardiovascular events, modulate tumour biology and amplify susceptibility to cancer therapy-related cardiovascular toxicity.

Within this framework, diet and lifestyle emerge as transversal modifiers of the MASLD–Cardio-Oncology Triangle. Mediterranean-style dietary patterns, individualised protein adequacy in sarcopenic obesity, optimisation of gut–liver axis homeostasis, judicious use of selected nutraceuticals and avoidance of energy-restrictive regimens in patients at risk of malnutrition collectively act on multiple nodes of this triangle. Although direct randomised evidence in oncology cohorts with documented MASLD is still limited, the convergence of mechanistic, epidemiological and clinical data supports the prospective evaluation of structured nutritional and metabolic assessment within cardio-oncology pathways (Figure 3).

For clinicians, current evidence supports awareness of MASLD as a systemic comorbidity, but routine incorporation into cardio-oncology risk scores cannot yet be recommended solely on the basis of available data. Within multidisciplinary care, hepatic phenotyping and nutritional assessment may be considered in selected patients, particularly those with metabolic risk factors. For the research community, the call-to-action is equally explicit: well-designed multicentre trials, standardised MASLD definitions, composite hepato-cardio-oncological endpoints and validated integrated risk scores are urgently needed to transform the conceptual framework proposed here into evidence-based clinical practice.

Recognising MASLD as a systemic disorder at the intersection of metabolism, cardiovascular biology and cancer represents, ultimately, an opportunity to evaluate whether integrated strategies can advance personalised survivorship care, reduce the long-term cardiovascular burden of oncological treatments and align nutritional, hepatological, cardiological and oncological strategies within a single, coherent and clinically actionable paradigm.

## Figures and Tables

**Table 1:** Hierarchy of evidence supporting the MASLD–Cardio-Oncology framework.

Topic	Current Level of Evidence	Key Message
MASLD and cardiovascular disease	Well-established clinical evidence	Supported by meta-analyses and international guidelines.
MASLD and extrahepatic cancer risk	Well-established clinical evidence	Consistent epidemiological associations, although the underlying mechanisms remain under active investigation.
MASLD and CTR-CVT	Emerging clinical evidence	Direct clinical evidence remains limited; prospective studies are needed to confirm the association and to define its magnitude.
Shared metabolic, inflammatory and mitochondrial pathways	Mechanistic rationale supported by experimental data	These pathways provide a biologically plausible explanation linking MASLD, CVD and cardio-oncology.
Hepatic phenotyping in cardio-oncology risk stratification	Translational hypothesis	Promising concept that requires prospective validation before clinical implementation.

*Abbreviations*: CVD, cardiovascular disease; CTR-CVT, cancer therapy-related cardiovascular toxicity; MASLD, metabolic dysfunction-associated steatotic liver disease.

## Data Availability

The original contributions presented in the study are included in the article; further inquiries can be directed to the corresponding authors.
